# Rab11 is required for lysosome exocytosis through the interaction with Rab3a, Sec15 and GRAB

**DOI:** 10.1242/jcs.246694

**Published:** 2021-06-08

**Authors:** Cristina Escrevente, Liliana Bento-Lopes, José S. Ramalho, Duarte C. Barral

**Affiliations:** iNOVA4Health, CEDOC, NOVA Medical School, NMS, Universidade NOVA de Lisboa, 1169-056 Lisboa, Portugal

**Keywords:** Sec15, Rab11, Rab3a, GRAB, RAB3IL1, Lysosome, Exocytosis

## Abstract

Lysosomes are dynamic organelles, capable of undergoing exocytosis. This process is crucial for several cellular functions, namely plasma membrane repair. Nevertheless, the molecular machinery involved in this process is poorly understood. Here, we identify Rab11a and Rab11b as regulators of Ca^2+^-induced lysosome exocytosis. Interestingly, Rab11-positive vesicles transiently interact with lysosomes at the cell periphery, indicating that this interaction is required for the last steps of lysosome exocytosis. Additionally, we found that the silencing of the exocyst subunit Sec15, a Rab11 effector, impairs lysosome exocytosis, suggesting that Sec15 acts together with Rab11 in the regulation of lysosome exocytosis. Furthermore, we show that Rab11 binds the guanine nucleotide exchange factor for Rab3a (GRAB) as well as Rab3a, which we have previously described to be a regulator of the positioning and exocytosis of lysosomes. Thus, our study identifies new players required for lysosome exocytosis and suggest the existence of a Rab11–Rab3a cascade involved in this process.

## Abstract (precis)

**Summary:** Rab11a and Rab11b, as well their effector Sec15, are required for lysosome exocytosis, and the Rab3a guanine nucleotide exchange factor GRAB is essential for this process.

## INTRODUCTION

Lysosomes are membrane-bound organelles that play a key role in the degradation of intracellular material. These organelles have long been regarded as the end-point of the endocytic pathway in eukaryotic cells. However, it is now recognized that there are distinct populations of lysosomes in the cell and that lysosomes in the perinuclear region are more acidic than lysosomes at the cell periphery (Johnson et al., 2016). Moreover, lysosomes that localize near the plasma membrane can undergo regulated exocytosis, similar to lysosome-related organelles (LROs) (Andrews, 2000; Rodríguez et al., 1997). In a first Ca^2+^-independent step, lysosomes translocate along microtubules from the perinuclear region to the cell periphery in a process that is dependent on BLOC-1-related complex (BORC)–Arl8–PLEKHM2–kinesin-1 (Pu et al., 2015; Rosa-Ferreira and Munro, 2011). In a second step, the pool of pre-docked lysosomes fuses with the plasma membrane, in response to an increase in intracellular Ca^2+^. This fusion requires the Ca^2+^-sensor synaptotagmin VII, as well as the R-soluble N-ethylmaleimide-sensitive factor (NSF) attachment protein receptor (R-SNARE) vesicle-associated membrane protein (VAMP)-7 on the lysosome membrane and the Q-SNAREs synaptosomal-associated protein 23 (SNAP23) and syntaxin-4 (STX4) on the plasma membrane (Martinez et al., 2000; Rao et al., 2004).

Lysosome exocytosis occurs in many cell types and is involved in a variety of cellular processes, including plasma membrane repair and extracellular matrix remodeling and/or degradation (Appelqvist et al., 2013; Hämälistö and Jäättelä, 2016; Jaiswal et al., 2002; Reddy et al., 2001; Samie and Xu, 2014). Plasma membrane repair is particularly important in tissues susceptible to mechanical or ischemic stress, such as skeletal and cardiac muscle (Cheng et al., 2014; Zhao, 2012). Extracellular matrix degradation is frequently enhanced in carcinomas following abnormal lysosomal secretion by cancer cells, and leads to stimulation of angiogenesis, tumor growth and cancer cell invasion (Appelqvist et al., 2013; Kallunki et al., 2013; Machado et al., 2015).

Although the molecular machinery involved in lysosome exocytosis is partially known, the role of Rab small GTPases in this process remains poorly understood. Recently, we described a screen to identify Rab proteins that regulate lysosome exocytosis (Encarnação et al., 2016). Rab3a, which is involved in regulated secretion, was one of the most-robust hits. Indeed, in lysosome positioning and plasma membrane repair we found that Rab3a plays an important role as part of a complex formed by the non-muscle myosin heavy chain IIA (encoded by *MYH9*, hereafter referred to as NMIIA) and synaptotagmin-like protein 4a (Sytl4, hereafter referred to as Slp-4a). Another hit was Rab11b, a Rab protein known to regulate endocytic recycling traffic (Ullrich et al., 1996; Welz et al., 2014), as well as the secretory pathway (Chen et al., 1998; Urbé et al., 1993). The Rab11 subfamily comprises Rab11a and Rab11b both of which are ubiquitously expressed, and Rab25 that is expressed in epithelial tissues of the gastrointestinal mucosa, kidney and lung (Goldenring et al., 1993; Kelly et al., 2012). Interestingly, previous studies have proposed a link between the endocytic recycling pathway and exocytosis in different cell types, including cytotoxic T-cells and bladder umbrella cells (Khandelwal et al., 2013; Ménager et al., 2007). Furthermore, our group established that Rab11b regulates the exocytosis of melanosomes, which are LROs (Tarafder et al., 2014). However, despite all evidence, the role of the endocytic recycling pathway and, in particular, the Rab11 subfamily in lysosome exocytosis remains poorly understood.

Similar to other small GTPases, Rab11 proteins cycle between active GTP-bound and inactive GDP-bound forms. GTP hydrolysis is catalyzed by GTPase-activating proteins (GAPs), whereas the exchange of GDP for GTP is promoted by guanine nucleotide exchange factors (GEFs) (Welz et al., 2014). In its active form, Rab11 recruits effector proteins. Among the known Rab11 effectors described in the literature are the Rab11-family of interacting proteins (Rab11FIPs) (Baetz and Goldenring, 2013; Hales et al., 2001; Horgan and McCaffrey, 2009; Junutula et al., 2004), motor proteins, such as myosinVa/b (Lapierre et al., 2001; Lindsay et al., 2013), and the octameric exocyst tethering complex subunit Sec15 (also known as EXOC6 in mammals) (Wu et al., 2005; Zhang et al., 2004). Moreover, Rab11 was found to interact with other proteins, including the Rab-3A-interacting protein Rabin8 (RAB3IP) (Bryant et al., 2010) and the GEF for Rab3a (RAB3IL1, also known as and hereafter referred to as GRAB) (Horgan et al., 2013).

In this study, we explored the role of Rab11a and Rab11b isoforms in Ca^2+^-triggered lysosome exocytosis. We show that the silencing of the small GTPases Rab11a or Rab11b impairs lysosome exocytosis, upon treatment with the Ca^2+^ ionophore ionomycin. Rab11-positive vesicles were found to transiently interact with lysosomes, as well as with late endosomes (LEs) at the cell periphery, indicating that this interaction is required for the last steps of LE/lysosome exocytosis. Moreover, silencing of Sec15 but not exocyst components Exo70, Sec8 or Sec10 (also known as EXOC7, EXOC8 or EXOC5, respectively), impairs lysosome exocytosis. Furthermore, we show that Rab11 co-immunoprecipitates with Rab3a, and silencing of GRAB − which interacts with Rab11 and is a Rab3a GEF – impairs lysosome exocytosis. Thus, our findings identify new players required for lysosome exocytosis.

## RESULTS

### Rab11a and Rab11b are required for Ca^2+^-dependent lysosome exocytosis

To identify Rab proteins that regulate Ca^2+^-dependent lysosome exocytosis, we performed a screen using lentiviral-expressed short hairpin RNA (shRNA) in THP1 monocytic cells, targeting 58 different Rab GTPases (Encarnação et al., 2016). The screening identified several Rab proteins potentially involved in lysosome exocytosis, including Rab3a and Rab10 (Encarnação et al., 2016; Vieira, 2016), and Rab11b. Rab11 proteins are known to regulate the endocytic recycling pathway (Ullrich et al., 1996; Welz et al., 2014), as well as the secretory pathway (Chen et al., 1998; Urbé et al., 1993). Interestingly, Rab11a and Rab11b were described to be involved in exocytosis of LROs but the molecular mechanism of this process is poorly understood (Ménager et al., 2007; Tarafder et al., 2014; Van der Sluijs et al., 2013).

**Fig. 1.**
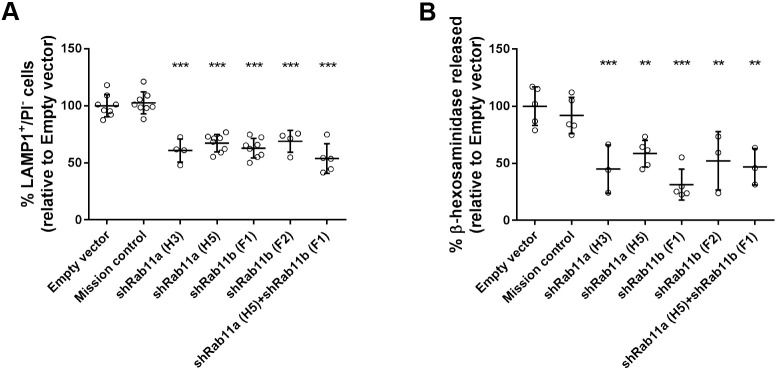
**Rab11a or Rab11b silencing impairs LAMP1 cell-surface translocation and β-hexosaminidase release.** HeLa cells transduced with lentiviruses encoding different shRNAs targeting Rab11a, Rab11b or both were treated with 10 μM ionomycin and 4 mM CaCl_2_ for 10 min at 37°C to trigger lysosome exocytosis. (A) Cells were collected, stained with anti-LAMP1 antibody and analyzed by flow cytometry. Plotted is the percentage of LAMP1-positive (LAMP1^+^) cells and propidium iodide-negative (PI^−^) cells. (B) Cell extracts and supernatants were collected and β-hexosaminidase release was quantified as described in the Materials and Methods. Cells transduced with an empty vector or a non-targeting shRNA (Mission) were used as negative controls. Results were normalized to empty vector and are represented as mean±s.d. of at least three independent experiments. ANOVA followed by Dunnett's multiple comparisons test was used to compare different data sets with empty vector (***P<0.001, **P<0.01).

To confirm whether Rab11 plays a role in Ca^2+^-dependent lysosome exocytosis, we silenced the isoforms Rab11a or Rab11b in HeLa cells and stimulated lysosome exocytosis with the Ca^2+^ ionophore ionomycin. Upon lysosome exocytosis, the lysosomal membrane fuses with the plasma membrane. Therefore, expression of the LE/lysosomal marker protein LAMP1 on the cell surface was measured by flow cytometry, using an antibody specifically recognizing the luminal domain of the protein. Non-viable cells were excluded by staining with propidium iodide (PI). We observed that HeLa cells that had been silenced for Rab11a or Rab11b by expressing specific shRNAs, show impaired cell-surface translocation of LAMP1 upon stimulation with ionomycin, compared with cells transduced with lentiviruses containing an empty vector (Empty) or encoding a non-targeting shRNA (Mission) (Fig. 1A). From the five shRNAs tested (Table S1), the four effectively silencing the protein yielded similar results (Fig. S1A) and, therefore, only two representative ones are shown. Quantitative real-time PCR (qRT-PCR) and western blotting confirmed that Rab11a and Rab11b were specifically and efficiently silenced (Fig. S1B-G).

Since LAMP1 is also present in LEs, we analyzed the release of the lysosomal hydrolytic enzyme β-hexosaminidase upon stimulation with ionomycin to ascertain whether Rab11a and Rab11b are specifically involved in lysosome exocytosis rather than in LE exocytosis only. We observed that cells depleted for Rab11a or Rab11b display significant impairment regarding the release of β-hexosaminidase when compared with cells transduced with empty vector or vector encoding non-targeting shRNA (Fig. 1B). Notably, HeLa cells transduced simultaneously with Rab11a and Rab11b shRNAs did not show increased impairment of lysosome exocytosis, suggesting that the function of Rab11a and Rab11b in lysosome exocytosis is not redundant (Fig. 1A,B).

Next, we overexpressed GFP-tagged Rab11a or Rab11b. Compared with cells transfected with only GFP-encoding vector, we did not find significant changes in LAMP1 cell-surface translocation levels, and only slightly increased release of β-hexosaminidase upon treatment with ionomycin (Fig. S2A,B). These results suggest that Ca^2+^-induced lysosome exocytosis cannot be further enhanced through overexpression of Rab11a or Rab11b, probably because there is a fixed lysosome pool that can undergo exocytosis. Surprisingly, HeLa cells overexpressing the constitutively active Rab11 mutants Rab11a Q70L or Rab11b Q70L, or the dominant-negative Rab11 mutants Rab11a S25N or Rab11b S25N did not show significant differences in the levels of LAMP1 cell-surface translocation or β-hexosaminidase release, when compared with cells overexpressing the wild-type form of the proteins (Fig. S2A,B). Thus, the Rab11 GTP-binding state does not seem to affect Ca^2+^-dependent lysosome exocytosis in HeLa cells.

### Interaction of Rab11-positive vesicles with LEs/lysosomes at the cell tips is enhanced after stimulation with ionomycin

**Fig. 2.**
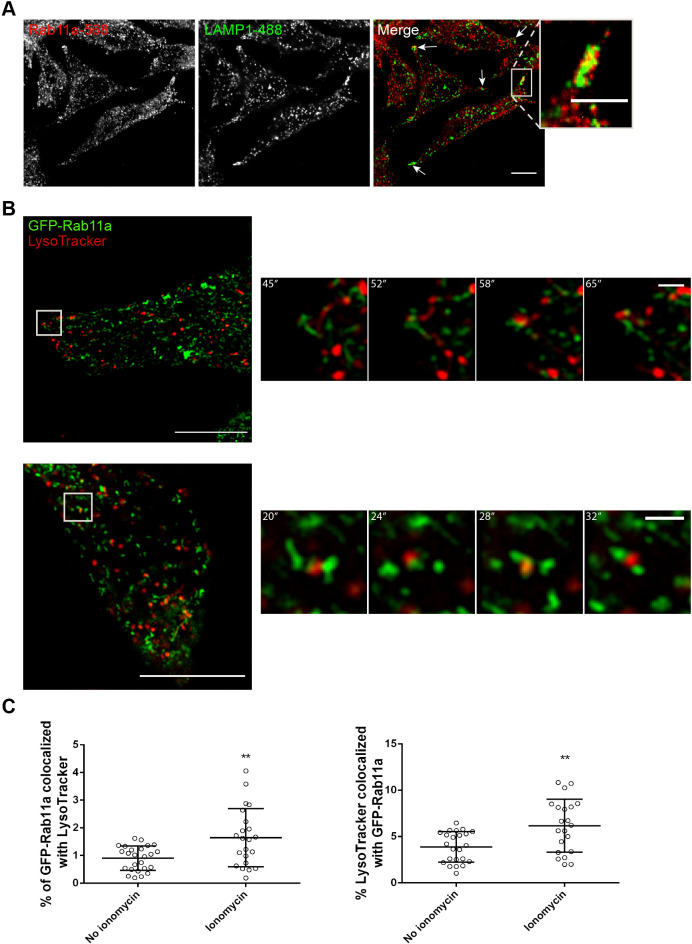
**Rab11a colocalization with** **LEs/lysosomes at the cell tips is enhanced upon** **stimulation with** **ionomycin.** (A) Representative confocal microscopy images of the intracellular localization of Rab11a and LAMP1, in HeLa cells, upon lysosome exocytosis stimulation with ionomycin. Cells were fixed and stained with rabbit anti-Rab11a (red) and mouse anti-LAMP1 (green) antibodies. White arrows indicate the areas at the cell tips where LAMP1 localizes in close proximity with Rab11a-positive vesicles. Scale bar: 10 μm. The boxed area shows an enlarged view of a cell tip. Scale bar: 5 μm. Results are representative of two independent experiments where at least 15 cells were analyzed per experiment. (B) Live cell imaging of HeLa cells transiently transfected with GFP-Rab11a (green) and incubated for 1–2 h with LysoTracker (red) to label LEs/lysosomes. The cells were imaged by super-resolution confocal microscopy immediately after stimulation with ionomycin. Images are from a *Z*-plane and were captured every 4–6 s for 60–90 s. Scale bar: 10 μm. The boxed area indicates a zoomed-in region of which consecutive frames taken at the indicated time points are shown. Scale bar: 1 μm. Results are representative of three independent experiments where at least eight cells per condition were analyzed per experiment. (C) The number of interactions observed between GFP-Rab11a-positive vesicles and LysoTracker-positive LEs/lysosomes was quantified in the absence of or immediately after incubation with ionomycin. Results are shown as mean percentage (%) of GFP-Rab11a colocalized with LysoTracker or of LysoTracker colocalized with GFP-Rab11a±s.d. of three independent experiments, where at least 21 cells were analyzed per condition. Unpaired two-tailed Student's *t*-test was used to compare differences between the two conditions (**P<0.01).

To gain insights into the molecular mechanism by which Rab11a and Rab11b regulate lysosome exocytosis, we analyzed the intracellular localization of Rab11a, Rab11b and LEs/lysosomes at steady-state levels and upon stimulation with ionomycin by using immunofluorescence confocal microscopy. When overexpressed, we observed a striking colocalization of GFP-tagged Rab11a and Rab11b (GFP-Rab11a and GFP-Rab11b, respectively) in HeLa cells (Fig. S3A). In addition, similar to the endogenous proteins, overexpressed GFP-Rab11a and GFP-Rab11b are distributed throughout the cytoplasm, with a striking accumulation at the perinuclear region. Furthermore, the LE/lysosome marker LAMP1 accumulates in the perinuclear region but also localizes throughout the cytoplasm and at the cell tips, confirming our previous observations (Encarnação et al., 2016). Upon stimulation with ionomycin, we observed that Rab11a-positive vesicles localize in close proximity with LEs/lysosomes, in particular at the cell tips (Fig. 2A). The same was observed for Rab11b (Fig. S3B). Thus, our results suggest that Rab11a- and Rab11b-positive vesicles interact with LEs/lysosomes at the cell periphery just before fusion with the plasma membrane. Furthermore, we observed that the silencing of Rab11a or Rab11b does not affect the distribution of LAMP1-positive vesicles in the cell (Fig. S3C).

It has been described that, upon stimulation, only a small percentage of lysosomes (∼10%) undergo exocytosis (Jaiswal et al., 2002; Rodríguez et al., 1997). Since this is a rare event, we used live cell imaging to detect transient interactions between Rab11-positive vesicles and lysosomes. For that, we transfected HeLa cells with GFP-Rab11a and tracked LEs/lysosomes labeled with LysoTracker, which stains acidic compartments. To understand the impact stimulation with ionomycin has on Rab11a and lysosome intracellular localization, we imaged HeLa cells by using super-resolution confocal microscopy before and immediately after stimulation with ionomycin (Fig. 2B, Fig. S4A, Movies 1 and 2). As observed in fixed cells, GFP-Rab11a accumulates at the perinuclear region, although it is also distributed throughout the cell. Similarly, LEs/lysosomes labeled with LysoTracker localize near the perinuclear region, but are also distributed throughout the whole cell. We also observed that LysoTracker-positive LEs/lysosomes transiently interact with GFP-Rab11a-positive vesicles (Fig. 2B, Fig. S4A, Movies 1–3). These events were mostly detected at the cell tips or close to the plasma membrane, and we obtained similar results upon overexpression of GFP-Rab11b (Movie 4). Quantification of the number of interactions showed a significant increase upon stimulation with ionomycin, when compared with cells without ionomycin (Fig. 2C). Furthermore, we found that GFP-Rab11a-positive vesicles partially colocalize with lysosomes labeled with dextran, particularly at the cell tips (Fig. S4B).

Altogether, these results suggest that the increase in intracellular Ca^2+^ concentration triggers the transient interaction of both lysosomes and LEs with Rab11-positive vesicles, near the cell periphery, before fusion with the plasma membrane.

### Sec15 is required for Ca^2+^-dependent lysosome exocytosis, independently of the exocyst tethering complex

**Fig. 3.**
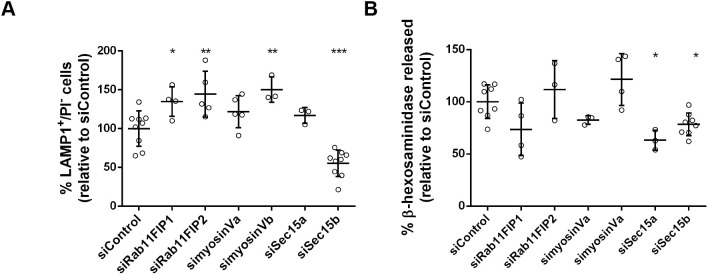
**Rab11 effector proteins are involved in lysosome exocytosis.** HeLa cells silenced for the indicated Rab11 effectors were treated with 10 μM ionomycin and 4 mM CaCl_2_ for 10 min at 37°C, to trigger lysosome exocytosis. (A) Cells were collected, stained with an anti-LAMP1 antibody and analyzed by flow cytometry. Plot represents the percentage of LAMP1-positive cells and propidium iodide (PI)-negative cells. (B) Extracts and supernatants of HeLa cells depleted of Rab11 effectors and treated with ionomycin were collected and β-hexosaminidase release was quantified as described in the Materials and Methods. Cells transfected with a non-targeting siRNA (siControl) were used as a negative control. Results were normalized to the siControl and are represented as mean±s.d. of at least three independent experiments. ANOVA followed by Dunnett's multiple comparisons test was used to compare different data sets with siControl (***P<0.001, **P<0.01, *P<0.05; all others are non-significant).

To further understand the role of Rab11 in Ca^2+^-dependent lysosome exocytosis, we searched for Rab11 effector proteins that mediate this function. To investigate whether any of the known Rab11 effectors are involved, we silenced the two class I Rab11FIP family members RAB11FIP1 and Rab11FIP2, postulated to regulate the movement of recycling vesicles (Baetz and Goldenring, 2013; Schafer et al., 2014). We also silenced myosinVa or myosinVb, which are involved in vesicle transport along the actin cytoskeleton (Lapierre et al., 2001; Lindsay et al., 2013), and the exocyst subunits Sec15a or Sec15b, which are part of the exocyst complex (Wu et al., 2005; Zhang et al., 2004). Silencing efficiency was confirmed by qRT-PCR (Fig. S1H); Ca^2+^-dependent lysosome exocytosis was evaluated by detecting LAMP1 at the cell surface, using flow cytometry (Fig. 3A), as well as by measuring release of β-hexosaminidase (Fig. 3B). Cells transfected with small interfering RNA (siRNA) not targeting specific genes (siControl) were used as a control. Among the Rab11 effectors tested, only Sec15 significantly reduced LAMP1 cell-surface translocation levels as well as β-hexosaminidase release, phenocopying the effect of Rab11a and Rab11b depletion. Curiously, LAMP1 cell-surface translocation levels only decreased in the absence of the Sec15b isoform, whereas release of β-hexosaminidase, decreased when silencing either Sec15a or Sec15b isoforms. Therefore, the Sec15a isoform seems to play a more-specific role in lysosome exocytosis, whereas Sec15b seems to regulate both LE and lysosome exocytosis. It is noteworthy that silencing of RAB11FIP1, RAB11FIP2 or myosinVb had the opposite effect, i.e. leading to increased LAMP1 cell-surface translocation levels (Fig. 3A). This suggests that these proteins negatively regulate LE/lysosome exocytosis. Silencing of myosinVa led to a noticeable decrease in β-hexosaminidase release, even though the difference was not statistically significant (Fig. 3B).

**Fig. 4.**
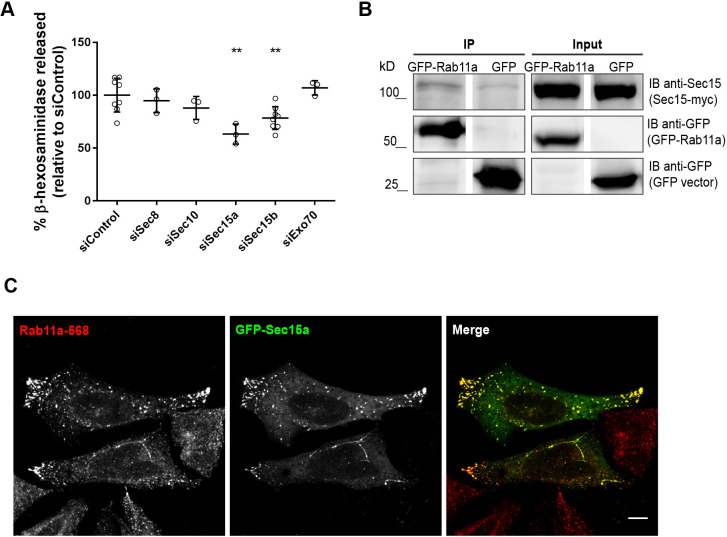
**Sec15 is required for lysosome exocytosis independently of the exocyst complex.** (A) To trigger lysosome exocytosis, HeLa cells silenced with siRNAs targeting the indicated exocyst subunits were treated with 10 μM ionomycin and 4 mM CaCl_2_ for 10 min at 37°C. Cell extracts and supernatants were collected and β-hexosaminidase release was quantified as described in the Materials and Methods. Cells transfected with a non-targeting siRNA (siControl) were used as controls. Results were normalized against those of siControl and are represented as mean±s.d. of at least three independent experiments. ANOVA followed by Dunnett's multiple comparisons test was used to compare different data sets with siControl (**P<0.01; all others are non-significant). (B) Co-immunoprecipitation of GFP-Rab11a with Sec15-myc in HeLa cells. Total cell extract (500 μg) was used to immunoprecipitate GFP-Rab11a. Cells expressing GFP were used as a negative control. Input corresponds to 1:10 of total cell extracts used for immunoprecipitation (50 μg). Western blotting was done using mouse anti-Sec15 and goat anti-GFP antibodies. Results are representative of two independent experiments. (C) Representative confocal microscopy images of HeLa cells overexpressing the exocyst subunit GFP-Sec15a (green) and stained with rabbit anti-Rab11a antibody (red). Scale bar: 10 µm. Results are representative of three independent experiments; at least 15 cells were analyzed per experiment.

Since we wanted to find effector proteins that mediate Rab11 function in lysosome exocytosis, we decided to further explore the role of Sec15, silencing of which has a similar effect on lysosome exocytosis than depletion of Rab11. Sec15 is part of the exocyst, a complex involved in tethering of vesicles including Rab11-positive recycling endosomes (Takahashi et al., 2012). To investigate whether the exocyst complex is involved in the tethering and fusion of lysosomes to the plasma membrane upon stimulation, we expressed siRNAs targeting different exocyst subunits in HeLa cells. We analyzed Sec8, a core component of the exocyst (Mei and Guo, 2018), Sec10, which forms a subcomplex with Sec15 in yeast and mammalian cells (Guo et al., 1999; Moskalenko et al., 2003), and Exo70, which mediates the association of the exocyst to the plasma membrane (He et al., 2007). Silencing efficiency was confirmed by qRT-PCR (Fig. S1H). Surprisingly, compared with siControl RNA-transfected cells, β-hexosaminidase release was not affected in cells transfected with Sec8-, Sec10- or Exo70-targeting siRNA (Fig. 4A), meaning only silencing of the Sec15 subunit impairs lysosome exocytosis. However, we observed, an albeit weak, co-immunoprecipitation of GFP-Rab11a with Myc-tagged Sec15a (Sec15-myc; Fig. 4B, Fig. S5A), as well with other exocyst subunits, i.e. Sec8 and Exo70 (Fig. S5B). This confirmed that Rab11 interacts with the exocyst tethering complex, as previously described by others (Takahashi et al., 2012). Next, we analyzed the intracellular localization of both Sec15a and Rab11. As expected, we found that GFP-tagged Sec15a prominently colocalizes with endogenous Rab11 (Fig. 4C). Moreover, Sec15a overexpression promoted endogenous Rab11 relocalization, increasing the number of Rab11-positive vesicles at cell tips (Fig. 4C). This result is in agreement with previous reports, showing that Rab11 effectors, namely Rab11FIPs, influence Rab11 localization when overexpressed (Baetz and Goldenring, 2013). Overall, our results suggest that Sec15, together with Rab11, plays an important role in Ca^2+^-dependent lysosome exocytosis.

### Rab11 and GRAB are required for Ca^2+^-dependent lysosome exocytosis

**Fig. 5.**
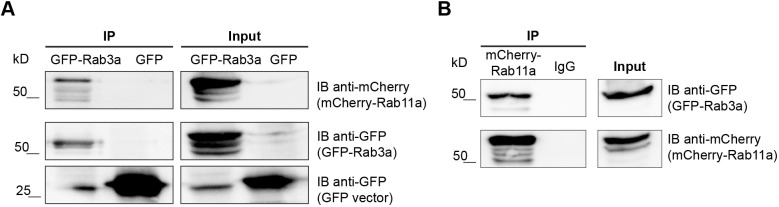
**Rab11 co-immunoprecipitates with Rab3a.** (A,B) Western blots of total cell extracts (500–600 μg) immunoprecipitated for GFP-Rab3a (A) or mCherry-Rab11a (B). Cells transfected with GFP-expressing vector only and goat IgG were used as negative controls. Western blotting was done using goat anti-mCherry or goat anti-GFP antibodies to detect mCherry-Rab11a or GFP-Rab3a. Input corresponds to 1:10 of total cell extracts used for immunoprecipitation. Results are representative of three independent experiments.

Upon stimulation with ionomycin, Rab11-positive vesicles transiently interact with LEs/lysosomes near the cell periphery, immediately before LE/lysosome fusion with the plasma membrane. We have recently shown that Rab3a silencing impairs lysosome exocytosis (Encarnação et al., 2016). Rab3a has also been shown to recruit effector proteins, namely Slp-4a and NMIIA, and to regulate lysosome positioning and exocytosis (Encarnação et al., 2016). Moreover, we observed that a small percentage of lysosomes localizing close to the cell periphery stain for Rab3a (Encarnação et al., 2016). This fraction of lysosomes is likely to correspond to the pool of lysosomes that fused with the plasma membrane upon the increase in intracellular Ca^2+^ concentration. Therefore, we hypothesized that Rab11 interacts with Rab3a on the surface of lysosomes. To test this hypothesis, we overexpressed GFP-tagged Rab3 (GFP-Rab3a) and mCherry-tagged Rab11a (mCherry-Rab11a) in HeLa cells, and performed co-immunoprecipitation studies. Upon immunoprecipitation of GFP-Rab3a, we detected a band corresponding to mCherry-Rab11a (Fig. 5A), indicating that these proteins do, indeed, interact. Co-immunoprecipitation of mCherry-Rab11a by using an anti-mCherry antibody followed by the detection of GFP-Rab3a (Fig. 5A) confirmed the interaction between these two Rab proteins.

**Fig. 6.**
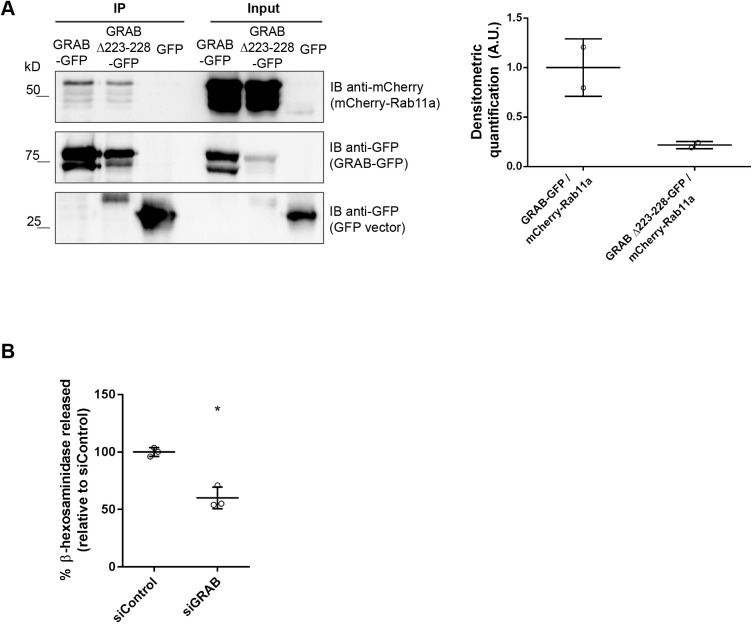
**GRAB interacts with Rab11 and is required for lysosome exocytosis.** (A) Total cell extracts (350 μg) were used to immunoprecipitate wild-type or mutant GRAB-GFP or GRAB Δ223-228-GFP. GFP immunoprecipitation was used as a negative control. Western blotting was done using goat anti-mCherry antibody to detect mCherry-Rab11a. Input corresponds to 1:10 of total cell extracts used for immunoprecipitation. Densitometric quantification (A.U., arbitrary unit) of the interaction between GRAB-GFP or GRAB Δ223-228-GFP with mCherry-Rab11a. Results were normalized to GRAB-GFP-mCherry-Rab11a interaction and are representative of two independent experiments. (B) HeLa cells were transfected with siRNA for GRAB. β-hexosaminidase release was evaluated as described in the Materials and Methods. Cells transfected with a non-targeting siRNA (siControl) were used as control. Results were normalized to the siControl and are represented as mean±s.d. of three independent experiments. Paired two-tailed Student's *t*-test was used to compare differences between siGRAB and siControl (*P<0.05).

To our knowledge, there is no precedent for Rab proteins interacting directly. We, therefore, postulated that the interaction between Rab11 and Rab3a is mediated by an adaptor protein. In the search for a protein that can link Rab3a and Rab11, we noticed that the Rab3a GEF GRAB (Luo et al., 2001) had been described to directly bind Rab11a and Rab11b (Horgan et al., 2013). Therefore, we investigated whether GRAB plays a role in lysosome exocytosis through Rab11a and Rab3a. We started by confirming the interaction between Rab11a and GRAB in HeLa cells. For that, we co-immunoprecipitated mCherry-Rab11a with GFP tagged to wild-type GRAB (GRAB-GFP) or to a mutant form of GRAB with impaired capacity to bind Rab11 (GRAB Δ223-228-GFP) (Horgan et al., 2013). As expected, mCherry-Rab11a co-immunoprecipitated efficiently with GRAB-GFP, but showed weaker binding to GRAB Δ223-228-GFP (Fig. 6A). This was confirmed by immunoprecipitation of mCherry-Rab11a (Fig. S6A).

Next, we investigated whether GRAB is required for lysosome exocytosis. To this end, we silenced GRAB by using an siRNA pool and monitored the release of β-hexosaminidase upon stimulation with ionomycin. Silencing efficiency was assessed using qRT-PCR (Fig. S6B). Interestingly, upon siRNA-mediated GRAB depletion we found a significant decrease in β-hexosaminidase release, whereas β-hexosaminidase release was unchanged at normal GRAB levels in response to transfection with siControl (Fig. 6B). This suggests that GRAB is also required for lysosome exocytosis, phenocopying Rab11 and Rab3a depletion. Furthermore, we were unable show a clear interaction between GFP-Rab3a or GRAB-GFP and Sec15-myc (Fig. S6C,D), indicating that Rab11 interaction with Sec15 is lost before Rab11 interacts with GRAB and Rab3a.

## DISCUSSION

Lysosome exocytosis is present in most cell types (Samie and Xu, 2014) and required for several biological functions, including plasma membrane repair and lysosomal secretion (Appelqvist et al., 2013; Hämälistö and Jäättelä, 2016; Jaiswal et al., 2002; Reddy et al., 2001; Samie and Xu, 2014). Although several reports attempted to dissect the molecular players involved in this pathway, the role of Rab small GTPases is not well established. Recently, we screened the Rab family of proteins for their requirement in lysosome exocytosis, and identified Rab3a and Rab10 as regulators of lysosome positioning and plasma membrane repair (Encarnação et al., 2016; Vieira, 2016). In the same screen, Rab11b – which has a known role in the regulation of endocytic recycling (Ullrich et al., 1996; Welz et al., 2014) as well as the secretory pathway (Chen et al., 1998; Urbé et al., 1993) – was found to be potentially involved in lysosome exocytosis. Moreover, we have shown that Rab11b is involved in the exocytosis of melanosomes, a type of LRO (Tarafder et al., 2014). More recently, Rab11a deficiency was shown to induce changes in LEs/lysosomes, leading to an increased number of acidic vesicles labeled with LysoTracker localized to the perinuclear region (Zulkefli et al., 2019).

In this current study, we confirmed and expanded the results obtained in the screen and show that Rab11a and Rab11b are key regulators of lysosome exocytosis in HeLa cells. Indeed, silencing of Rab11a or Rab11b isoforms significantly impairs the exocytosis of lysosomes in response to Ca^2+^ stimulation. Interestingly, the reduction in lysosome exocytosis is not enhanced further when both Rab11 isoforms are silenced simultaneously, suggesting that both Rab11 isoforms are important but their function is not redundant. We attempted to restore lysosome exocytosis in cells depleted for Rab11a or Rab11b by overexpressing the same Rab isoform but observed that silenced cells overexpressing GFP display increased levels of both LAMP1 cell-surface translocation and β-hexosaminidase release, thus precluding this approach.

To investigate the mechanism by which Rab11a and Rab11b proteins are involved in lysosome exocytosis, we used live cell imaging to track Rab11-positive vesicles and LEs/lysosomes. Upon stimulation with ionomycin, we observed that Rab11-positive vesicles interact transiently with LEs/lysosomes near the cell periphery. We postulate that this interaction is important for the delivery of molecules required for the last steps of lysosome tethering and fusion with the plasma membrane. Studies in cytotoxic T cells have already shown the existence of transient interactions between immature lytic granules and Rab11-positive vesicles, suggesting they are essential to prime lytic granules during the last steps of exocytosis (Ménager et al., 2007; Van der Sluijs et al., 2013). Therefore, in the absence of Rab11, lysosomes might lack some of the machinery required for efficient exocytosis, leading to lysosome secretion impairment.

Similar to other Rab GTPases, Rab11 regulates vesicular trafficking by recruiting effector proteins. Therefore, we searched for Rab11 effectors that play a role in lysosome exocytosis. Although Rab11 has several known effector proteins (Baetz and Goldenring, 2013; Hales et al., 2001; Horgan and McCaffrey, 2009; Junutula et al., 2004; Lapierre et al., 2001; Lindsay et al., 2013; Wu et al., 2005; Zhang et al., 2004), we observed that, from those tested, only silencing of the exocyst subunit Sec15 leads to a consistent decrease in LAMP1 cell-surface translocation and β-hexosaminidase release, phenocopying Rab11 depletion. Additionally, confocal immunofluorescence microscopy confirmed that GFP-Sec15 colocalizes with endogenous Rab11. This suggests that Sec15 acts together with Rab11 in the regulation of lysosome exocytosis. Sec15 is part of the exocyst complex, which is implicated in the tethering of secretory vesicles to the plasma membrane (Heider and Munson, 2013) and exocytosis of recycling endosomes (Takahashi et al., 2012). Recently, our group has also demonstrated that the exocyst complex, together with Rab11b, is required for melanosome exocytosis (Moreiras et al., 2019). Surprisingly, the silencing of other exocyst subunits does not affect lysosome exocytosis. Few studies have addressed the question of whether exocyst subcomplexes or subunits can perform functions independently of the exocyst complex (Mehta et al., 2005; Moskalenko et al., 2003; Singh et al., 2019). Our results suggest that the role of Sec15 in lysosome exocytosis is independent of the exocyst complex. Nevertheless, we cannot exclude that other exocyst subunits or subcomplexes play a role in lysosome exocytosis.

Although the precise role of Sec15 in lysosome exocytosis is not entirely clear, it is likely to act as an adaptor protein between Rab11-positive vesicles and other molecules, for example myosins. This would be important for the efficient trafficking of Rab11-positive vesicles to the cell periphery, where they interact with lysosomes and prime them for exocytosis. In fact, Sec15 interacts with the myosinVa orthologue Myo2 of yeast (Jin et al., 2011) and *Candida albicans* (Guo et al., 2016). Furthermore, Sec15 participates in the transport of secretory vesicles by acting as an adaptor between Myo2 and the Rab11 orthologue Ypt31/32 or the Rab8/10 homologue Sec4 of yeast (Jin et al., 2011). A similar mechanism might occur in mammalian cells. Interestingly, we found decreasing secretion of β-hexosaminidase upon silencing of myosinVa in HeLa cells.

**Fig. 7.**
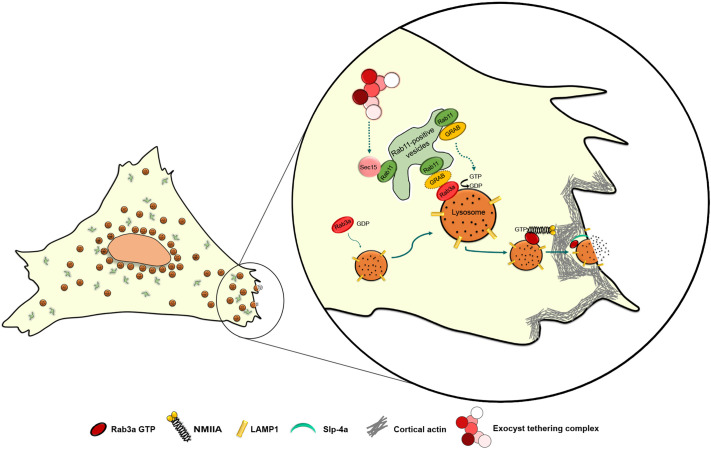
**Proposed** **mechanism for the role of Rab11 in** Ca^2+^**-triggered lysosome exocytosis.** Rab11 binds directly to Sec15, a subunit of the exocyst complex. Sec15 might function independently of the exocyst complex, facilitating the transport of Rab11-positive vesicles along the actin cytoskeleton via the interaction with myosin V (not shown). Rab11-positive vesicles are derived from the endocytic recycling compartment (ERC) or the Golgi (post-Golgi vesicles). Rab11-positive vesicles interact with Rab3a-positive lysosomes, possibly to deliver cargo proteins required for lysosome exocytosis, e.g. GRAB. We hypothesize that GRAB mediates the interaction between Rab11 and Rab3a. GRAB could then catalyze the conversion of Rab3a from a GDP-bound to GTP-bound state, thereby activating it, which recruits NMIIA and Slp-4a, allowing the correct positioning of lysosomes before lysosome exocytosis.

Since both Rab3a and Rab11 regulate lysosome exocytosis, we investigated whether they interact. Indeed, by co-immunoprecipitation, we found that Rab11 interacts with Rab3a. We, therefore, hypothesize that Rab11-positive vesicles interact with the subset of Rab3a-positive lysosomes that localizes at the cell periphery and are prone to fuse with the plasma membrane. Although we cannot discard a direct interaction between Rab11 and Rab3a, Rab proteins usually interact via adaptor proteins. By searching the literature for molecules that could link the two Rab small GTPases, GRAB became an obvious candidate, since it can interact directly with Rab11 (Horgan et al., 2013), and also is a Rab3a GEF (Luo et al., 2001). As hypothesized, we found that silencing of GRAB leads to decreased release of β-hexosaminidase and impairment of lysosome exocytosis, phenocopying Rab11 and Rab3a depletion. Therefore, we propose that GRAB binds to Rab11 and is transported by Rab11-positive vesicles to the vicinity of Rab3a-positive lysosomes, where it acts as a Rab3a GEF, converting Rab3a to its active form (Fig. 7). Interestingly, a similar cascade, involving Rab27–GRAB–Rab3a has recently been described to regulate human sperm exocytosis (Quevedo et al., 2019). Furthermore, since we could not detect any obvious interaction between GRAB or Rab3a and Sec15, it is possible that Rab11 interacts with GRAB and Rab3a after its interaction with Sec15, therefore suggesting that Sec15 plays a role in a different step of the lysosome exocytosis process (Fig. 7). Future studies might elucidate this hypothesis further.

Thus, our study provides valuable insights into the role of Rab11 in Ca^2+^-triggered lysosome exocytosis. Moreover, the knowledge acquired sheds light on the molecular mechanism behind several human disorders that affect plasma membrane repair and exocytosis of LROs.

## MATERIALS AND METHODS

### Cell culture

HeLa and HEK293T cells were maintained at 37°C and 5% CO_2_, in Dulbecco's modified Eagle medium (DMEM, Invitrogen) supplemented with 10% fetal bovine serum (FBS, Invitrogen), 100 U/ml penicillin G, 100 mg/ml streptomycin, 2 mM L-glutamine and 20 mM HEPES (Invitrogen). Both cells lines were obtained from the Division of Rheumatology, Immunology and Allergy, Brigham and Women's Hospital, Harvard Medical School. Neither cell line had recently been authenticated or tested for contamination.

### Cell transduction

Lentiviruses were produced in HEK293T cells in accordance with BL-2+ protocols publicly available at the Broad Institute's RNAi Consortium website. VSV-G, pLKO.1 and pCMV-dR8.91 plasmids were obtained from the RNAi Consortium. The shRNA sequences are listed in Table S1. Controls used were an empty vector and a non-targeting sequence (Mission; 5′-CAACAAGATGAAGAGCACCAA-3′; Sigma).

For lentiviral transduction, HeLa cells plated in six-well plates were incubated with shRNA-encoding lentivirus supernatants in the presence of 6 μg/ml polybrene (hexadimethrine bromide; Sigma-Aldrich). Twenty-four hours after infection, virus-containing medium was replaced with selection medium containing 2 µg/ml puromycin (Sigma). All assays were performed 7–8 days after infection.

### Cell transfection

Gene silencing of Rab11 effector proteins was performed with siGENOME SMARTpool oligonucleotides (Thermo Scientific Dharmacon) using DharmaFECT 1 (Dharmacon), according to the manufacturer's instructions. Briefly, HeLa cells, plated in 24-well plates, were incubated with a transfection mixture containing 40 nM of siRNA and 2 µl of transfection reagent. Assays were performed 72–96 h after siRNA transfection. The list of siRNA sequences is described in Table S2. As a control, a non-targeting siRNA sequence, siControl (5′-UAAGGCUAUGAAGAGAUAC-3′; Thermo Scientific Dharmacon) was used.

Plasmid transfections were performed using Lipofectamine 2000 (Invitrogen) in accordance with the manufacturer's instructions. Briefly, HeLa cells, plated in 24-well plates, 10-cm dishes or Nunc™ Lab-Tek™ Chambered Coverglasses (Thermo Fisher Scientific) were incubated with 1 µg DNA/2 µl Lipofectamine, 10 µg DNA/20 µl Lipofectamine or 0.5 µg DNA/1 µl of Lipofectamine, respectively. Experiments were performed 24–48 h after transfection. Plasmids used are described in Table S3.

### RNA extraction, cDNA production and quantitative real-time PCR

Total RNA was prepared with RNeasy Mini Kit (Qiagen), according to the manufacturer's instructions. Reverse transcription into complementary DNA (cDNA) was achieved by incubating 1 µg of total RNA with 1 mM of dNTP mix (Thermo Fisher Scientific) and 0.3 µg/µl of random primers p(DN) (Roche) at 65°C for 5 min, followed by incubation with 2× first strand buffer (Invitrogen), 20 mM DTT (Invitrogen) and 40 U/µl of Recombinant Ribonuclease Inhibitor RNaseOUT (Invitrogen) at 25°C for 2 min. Finally, 50 U/µl of Superscript II Reverse Transcriptase (Invitrogen) was added, samples were incubated at 25°C for 10 min, 42°C for 50 min and, finally, at 70°C for 15 min. Quantitative real-time PCR (qRT-PCR) was performed using the Fast Start Essential DNA Green Master (Roche) kit according to the manufacturer's instructions. Analysis was done in a qPCR Roche Light Cycler. β-actin was used as an endogenous control to normalize the expression level of each gene analyzed. A complete list of primer sequences used for qRT-PCR is provided in Table S4.

### Ca^2+^-induced lysosome exocytosis assay

Lysosome exocytosis was induced in HeLa cells using the Ca^2+^ ionophore, ionomycin (Sigma-Aldrich), as previously described (Encarnação et al., 2016). Briefly, HeLa cells were incubated in Ca^2+^- and Mg^2+^-free ice-cold Hanks Balanced Salt Solution (HBSS, Gibco) with 2.5–10 µM ionomycin in the presence of 4 mM CaCl_2_, for 10 min at 37°C. Cells incubated with HBSS alone were used as a control. After incubation, cells were placed on ice and processed for flow cytometry, β-hexosaminidase release assay or confocal cell microscopy.

### Flow cytometry

Cells incubated in HBSS, with or without 10 µM ionomycin and 4 mM CaCl_2_, were placed on ice and detached with flow cytometry buffer (PBS, 1% FBS and 2 mM EDTA). Cells were stained with an antibody that recognizes the luminal epitope of the human LAMP1 protein (clone H4A3, BioLegend) conjugated to Alexa Fluor 488 or Alexa Fluor 647 for 30 min at 4°C. To exclude dead cells, samples were incubated with 0.5 µg/ml of propidium iodide (PI) (Sigma-Aldrich) just before acquisition. Acquisition was performed in a FACS Calibur or FACS CANTO II flow cytometer and at least 30,000 cells were analyzed using FlowJo version 10.1r7 software.

### β-hexosaminidase release assay

Cells incubated in HBSS with or without 10 µM ionomycin and 4 mM of CaCl_2_, were placed on ice and cell supernatants were collected. In parallel, cells were lysed with 1% IGEPAL and further diluted 1:5 in dH_2_O. β-hexosaminidase activity was determined by incubating cell supernatants or lysates in a 96-well plate with 6 mM of the substrate 4-methylumbelliferyl- N-acetyl-β-d-glucosaminide (4-MU-β-D-GlcNAc; Glycosynth) resuspended in HBSS with 40 mM sodium citrate and 88 mM Na_2_PO_4_ pH 4.5 for 15 min at 37°C. Fluorescence was measured in a plate spectrofluorimeter Infinite F200 Pro reader (Tecan) at 365 nm excitation and 450 nm emission. The protein content from cell supernatants and lysates was determined simultaneously by using the BCA protein assay kit (Pierce Laboratories) as described by the manufacturer. Absorbance was measured at 560 nm in the same plate reader. HBSS and 1% IGEPAL diluted 1:5 were used as controls. β-hexosaminidase (β-hex) activity was calculated for each sample normalizing to total protein amount as following: β-hex activity in supernatant=(fluorescence (365/450)−HBSS alone)/protein µg. β-hex activity in cell lysate=(fluorescence (365/450)−IGEPAL alone)/protein µg. Total β-hex activity=β-hex activity in supernatant+5× β-hex activity in cell lysate. Finally, the percentage of β-hex release was calculated as following: β-hex release (% of total)=100×(β-hex activity in the supernatant/total β-hex activity).

### Confocal microscopy and live cell imaging

HeLa cells, grown on coverslips, were incubated in HBSS with or without 2.5 µM ionomycin and 4 mM of CaCl_2_. Cells were fixed for 15 min in 4% paraformaldehyde (Alfa Aesar) and blocked/permeabilized for 30 min in PBS containing 1% BSA and 0.05% saponin. Cells were then incubated with primary antibodies rabbit anti-Rab11a (Abcam) (1:500), rabbit anti-Rab11b (Abgent) (1:100), mouse anti-LAMP1 conjugated to Alexa Fluor 488 (BioLegend) (1:500) for 1 h, followed by incubation with Alexa Fluor 488- or Alexa Fluor 568-conjugated goat anti-rabbit secondary antibodies (1:1000) (Invitrogen). Cells were further incubated with 1 µg/ml DAPI (Sigma) and mounted in Mowiol mounting medium (Calbiochem). Images were acquired on a Zeiss LSM 710 confocal microscope with a Plan-Apochromat 63×1.4 NA oil-immersion objective. Digital images were analyzed with LSM Image software or ImageJ. Images represent the results of three independent experiments where at least 15 cells were analyzed in each experiment.

For live-cell confocal imaging, HeLa cells were seeded in Nunc™ Lab-Tek™ Chambered Coverglasses (Thermo Fisher Scientific) and transfected with GFP-Rab11a, as previously described. Twenty-four hours after transfection, cells were incubated with 50 nM LysoTracker^®^ Red DND-99 (Invitrogen) for 1–2 h. After extensive washing with PBS, cells were kept in Phenol Red-free DMEM (Gibco) for imaging. Cells were imaged before and after stimulation with 4 µM ionomycin and 4 mM CaCl_2_. Live-cell imaging was performed in HBSS at 37°C, using a Zeiss LSM 980 Airyscan 2 confocal microscope with a Plan-Apochromat 63×1.4 DIC f/ELYRA oil-immersion objective in the Multiplex SR-4Y imaging mode or an Andor Revolution spinning disk confocal microscope (Andor Technology) equipped with an EMC CD camera with a Plan Apo VC PFS 60×1.4 NA oil-immersion objective and controlled by iQ software (Andor Technology). Three independent experiments were performed and at least eight cells per condition were analyzed in each assay, using ImageJ software.

Interactions observed between GFP-Rab11a-positive vesicles and LysoTracker-positive LEs/lysosomes, in the absence or immediately after stimulation with ionomycin, were identified by using the spot detector plugin of ICY software. Spot colocalization, according to size and sensitivity, was calculated using the Ripley analysis and the statistical method SODA (Statistical Object Distance Analysis), considering the maximal distance of five pixels. Each dot represents the mean value of the colocalizations detected in all the time frames of each video image and results are shown as the mean percentage of GFP-Rab11a colocalized with LysoTracker or of LysoTracker colocalized with GFP-Rab11a±s.d.

### Immunoprecipitation

HeLa cells were lysed in ice-cold RIPA lysis buffer (50 mM Tris-HCl pH 7.5, 1 mM EDTA, 1 mM EGTA, 150 mM NaCl, 2 mM MgCl_2_, 1 mM DTT, 1% IGEPAL), in the presence of protease and phosphatase inhibitors, for 30 min, at 4°C. After centrifugation at 18,800× ***g*** for 30 min, at 4°C, protein concentration was determined using the DC protein assay kit (Bio-Rad), according to the manufacturer's instructions.

Proteins fused with GFP were immunoprecipitated for 2 h at 4°C, using GFP-Trap Beads (ChromoTek), previously equilibrated in 150 mM NaCl RIPA, using 350–800 µg of total cell extracts. Samples were centrifuged at 2500× ***g*** for 3 min at 4°C and washed twice with RIPA containing 500 mM NaCl, and three times with RIPA containing 150 mM NaCl. Finally, samples were solubilized in 2× Laemmli sample buffer and boiled at 95°C for 5 min.

Immunoprecipitation of endogenous and mCherry-fused proteins was performed using 500–700 µg of total protein pre-cleared for 1 h with Protein G-Sepharose beads (GE Healthcare Life Sciences). Cell lysates were incubated overnight, at 4°C, with 2 µg of goat anti-mCherry (Sicgen) or rabbit anti-Rab11a (Abcam) antibodies. The same concentration of goat IgG (Invitrogen) or rabbit IgG_1_ (Sigma) were used as negative controls. ProteinG-Sepharose beads were then added and incubated for 5 h at 4°C, under constant agitation. Beads were recovered by centrifugation, washed twice in RIPA containing 500 mM NaCl, and three times in RIPA with 150 mM NaCl. Finally, samples were solubilized in 2× Laemmli sample buffer and boiled for 5 min at 95°C.

### Western blotting

Proteins were loaded on 8–10% SDS-polyacrylamide gels, transferred to Nitrocellulose membranes (GE Healthcare Life Sciences) for 60 min at 100 V and processed for western blotting. Membranes were blocked with blocking buffer (5% milk in PBS with 0,1% Tween-20) and incubated with antibodies diluted in the same buffer. Primary antibodies used are described in Table S5. Horseradish peroxidase (HRP)-conjugated secondary antibodies (GE Healthcare) used were anti-mouse/rabbit (1:5000) or anti-goat antibodies (1:10,000) (Amersham ECL goat, rabbit or mouse IgG, HRP-linked whole Ab, GE Healthcare Life Sciences). HRP activity was detected with Amersham ECL Select (GE Healthcare Life Sciences), according to the manufacturer's instructions. Chemiluminescence was detected using a ChemiDoc™ Touch Imaging and the results were analyzed using ImageLab software.

### Statistical analysis

Numerical data are presented as mean±standard deviation (±s.d.). Normal distribution of the data was determined using the Shapiro-Wilk normality test. One-way ANOVA followed by Dunnett's multiple comparisons test was used to compare different data sets with a control and paired two-tailed Student's *t*-test was used to compare differences between two groups. Statistical analysis was performed using GraphPad Prism version 7.00.

## Supplementary Material

Supplementary information

Reviewer comments
