# Supplementary material for: Rab11 is required for lysosome exocytosis through the interaction with Rab3a, Sec15 and GRAB
Source: J Cell Sci. 2021 Jun 8;134(11):jcs246694. doi: 10.1242/jcs.246694 (PMC8214760; doi:10.1242/jcs.246694)
Supplement: Supplementary information [file joces-134-246694-s1.pdf]

**Table S1.** shRNAs used to transduce HeLa cells.

| Gene name | Accession number | Hairpin number | Source/Clone ID | Target sequence (5′–3′) |
|---|---|---|---|---|
| ***RAB11A*** | NM_004663 | H2 | TRCN0000073018 | CCC TAT AAA CAT AGC AGG ATT |
| ***RAB11A*** | NM_004663 | H3 | TRCN0000073019 | CGA GCT ATA ACA TCA GCA TAT |
| ***RAB11A*** | NM_004663 | H4 | TRCN0000073020 | CCT GTC TCG ATT TAC TCG AAA |
| ***RAB11A*** | NM_004663 | H5 | TRCN0000073021 | GAG CTA TAA CAT CAG CAT ATT |
| ***RAB11A*** | NM_004663 | H6 | TRCN0000073022 | GCC TTA TTG GTT TAT GAC ATT |
| ***RAB11B*** | NM_004218 | F1 | TRCN0000029184 | GCC TTG GAT TCC ACT AAC GTA |
| ***RAB11B*** | NM_004218 | F2 | TRCN0000029185 | CCT ATT CAA AGT GGT GCT CAT |
| ***RAB11B*** | NM_004218 | F3 | TRCN0000029186 | CCA CTA ACG TAG AGG AAG CAT |
| ***RAB11B*** | NM_004218 | F4 | TRCN0000029187 | CGT AGA GGA AGC ATT CAA GAA |
| ***RAB11B*** | NM_004218 | F5 | TRCN0000029188 | CAA GCA CCT GAC CTA TGA GAA |
| ***RAB11B*** | NM_004218 | F5 | TRCN0000029188 | CAA GCA CCT GAC CTA TGA GAA |

Journal of Cell Science • Supplementary information

**Table S2**. siRNAs used to silence Rab11 effectors.

| Gene name | Accession number | Target sequences |
|---|---|---|
| ***RAB11FIP1*** | NM_001002814 | CAAACAGAAGGAAACGAUA<br>GCUAACUGCAGCUUGGGAA<br>AGUGAGAACUUGAACAAUG<br>UCCGCGAGCUGGAAGACUA |
| ***RAB11FIP2*** | NM_001330167 | GGAUGAAGGUGAAUUGUGU<br>CGAGCUACCUGGAUUGCUA<br>GGAACAAUAUGACCGCAAG<br>GAUAAGAUGAAGGGUAGAA |
| ***MYO5A*** | NM_000259 | GAACAAAUGUGCACUCUUU<br>GAUCAUCUGCUCUGGAUUA<br>AAAGUAAGGUCGUUGCUAA<br>CGCAGGAGGUACAAGAUUA |
| ***MYO5B*** | NM_001080467 | GGACUUACCUCUUGGAGAA<br>CAAGUUGGCCUAACAGUGA<br>GCAGAUCUGGCCUACAAUA<br>ACAGUGGCCUUUAUACGAA |
| ***SEC8 (EXOC4)*** | NM_021807 | GAAUUGAGCAUAAGCAUGU<br>UAACUGAGUACUUGGAUAU |

Journal of Cell Science • Supplementary information

| | | |
|---|---|---|
| | | GCCGAGUUGUGCAGCGUAA |
| | | ACUGAGUGACCUUCGACUA |
| ***SEC10 (EXOC5)*** | NM_006544 | GAAGUCCGAUGCAGAGCAA |
| | | GGAGAUACCUUAUGACACA |
| | | GGAAAGAAUUAGACAGCGU |
| | | CAUUAGGAGUGGAUCGGAA |
| ***SEC15A (EXOC6)*** | NM_019053 | GAAGUUUGGUGAAUGGUAU |
| | | GUUGAUGGCUAUAGAAGAU |
| | | GAUAGAGACAGUCGUGAAA |
| | | CCAAACUCCGUGAGGAUAU |
| ***SEC15B (EXOC6B)*** | NM_015189 | UAACUGAACUGCUGAAAGU |
| | | GUACUAGUCCGAAGUCUGA |
| | | CAAGUAAGCCACUAUCGAU |
| | | CGGGAAACAUUUGAGAAUU |
| ***EXO70 (EXOC7)*** | NM_001013839 | GGUUAAAGGUGACUGAUUA |
| | | GACCUUCGACUCCCUGAUA |
| | | CUAAGCACCUAUAUCUGUA |
| | | CGGAGAAGUACAUCAAGUA |
| ***GRAB (RAB3IL1)*** | NM_013401 | GAGAGAAGGGCUCCGAGUU |
| | | GGCCUGGACUUCACGGCAA |
| | | AGAUCAUGAGGUUGCGGAA |
| | | ACAUGAAGCAGGCGGCAUC |

Journal of Cell Science • Supplementary information

**Table S3.** DNA plasmids used for transfection.

| Protein | Plasmid | Source |
|---|---|---|
| **GFP** | pENTR-GFPC1 | José Ramalho (Sicgen) |
| **mRab11a** | pENTR-GFPC1 | José Ramalho (Sicgen) |
| **mRab11b** | pENTR-GFPC1 | José Ramalho (Sicgen) |
| **mRab11a S25N** | pENTR-GFPC1 | José Ramalho (Sicgen) |
| **mRab11a Q70L** | pENTR-GFPC1 | José Ramalho (Sicgen) |
| **mRab11b S25N** | pENTR-GFPC1 | José Ramalho (Sicgen) |
| **mRab11b Q70L** | pENTR-GFPC1 | José Ramalho (Sicgen) |
| **mCherry** | pCDNA-ENTR-BP-mCherry | José Ramalho (Sicgen) |
| **mRab11a** | pCDNA-ENTR-BP-mCherry | José Ramalho (Sicgen) |
| **mRab11b** | pCDNA-ENTR-BP-mCherry | José Ramalho (Sicgen) |
| **mRab3a** | pENTR-GFPC2 | José Ramalho (Sicgen) |
| **rSec15** | pJ3 Myc-EGFP | Kind gift of Wei Guo |
| **rSec15** | pCDNA3 myc | Kind gift of Wei Guo |
| **hGRAB** | pGFP | Kind gift of Mary McCaffrey |
| **hGRABΔ223-228** | pGFP | Kind gift of Mary McCaffrey |

Journal of Cell Science • Supplementary information

**Table S4.** Primers used for qRT-PCR.

| Target gene | Sequence |
|---|---|
| *B-ACTIN* | Forward 5'-GCAAAGACCTGTACGCCAAC-3' |
| | Reverse 5'-AGTACTTGCGCTCAGGAGGA-3' |
| *RAB11A* | Forward 5'-CGATGGCTGAAAGAACTGAG-3' |
| | Reverse 5'-GACAGCACTGCACCTTTGGC-3' |
| *RAB11B* | Forward 5'-TCACCCGCAACGAGTTCAAC-3' |
| | Reverse 5'-CTGCACCACGGTAGTACGC-3' |
| *RAB11FIP1* | Forward 5'-CAAGGAGCGAGGAGAAATTG-3' |
| | Reverse 5'-GGTGTCTGACCCACTGTCCT-3' |
| *RAB11FIP2* | Forward 5'-TGGGGGATCTGATAGCCCTT-3' |
| | Reverse 5'-ACTCATATGAAAACTTGAAGATGGC-3' |
| *SEC8* | Forward 5'-ATGGCCAGCAAGCACTATCT-3' |
| | Reverse 5'-AGGTGCCGGTGTAGTTCATC-3' |
| *SEC10* | Forward 5'-ATAAAGCAGTGCCAGGAGGG3' |
| | Reverse 5'-AGCCAGGACTGTTTCTGGATT3' |
| *SEC15A* | Forward 5'-GTCACTACACCGGAGCTCAA3' |
| | Reverse 5'-TGCTCCAGGTGTGTTGTGTT3' |
| *SEC15B* | Forward 5'-CTGACCCTGCTTGAGAAGATGA3' |

Journal of Cell Science • Supplementary information

| | |
|---|---|
| | Reverse 5’-GCCACGGTGTCAATGAGTTTC-3’ |
| *EXO70* | Forward 5’-CATGGGTTATCAGGGGATTTG-3’ |
| | Reverse 5’-GAGGTCCAGGTGTGGGTAGA-3’ |
| *MYO5A* | Forward 5’-CAGTGGTCAGAACATGGGTG-3’ |
| | Reverse 5’-TCGCATGGCATACTTAGCTG-3’ |
| *MYO5B* | Forward 5’-AGAACTGGAGGAGGAGCGAT-3’ |
| | Reverse 5’-GGTTTGATGGGTTCCGCCTA-3’ |
| *GRAB* | Forward 5’-ATCCGCTACATCCAGCAAGG3’ |
| | Reverse 5’-TAAGCCTCCTGGGGGAAGAA3’ |

Journal of Cell Science • Supplementary information

**Table S5**. Antibodies used for immunoprecipitation and immunoblotting.

| Primary antibodies | Host animal | Immunoblotting dilution |
|---|---|---|
| **Rab11a (Abcam) (ab128913)** | Rabbit | 1:50,000 |
| **Rab11b (Abgent) (AP12943b)** | Rabbit | 1:1,000 |
| **Sec8 (Enzo Life Sciences) (ADI-VAM-SV016-F)** | Mouse | 1:500 |
| **Sec15 (Sigma) (SAB4200612)** | Mouse | 1:1,000 |
| **Exo70 (Milipore) (MABT186)** | Mouse | 1:1,000 |
| **GFP (Sicgen) (AB0020-200)** | Goat | 1:1,000 |
| **mCherry (Sicgen) (AB0040-200)** | Goat | 1:1,000 |
| **GFP (NeuroMab) (clone N86/8)** | Mouse | 1:3,000 |
| **Goat IgG Isotype Control (Invitrogen) (026202)** | Goat | - |

| | | |
|---|---|---|
| **Rabbit IgG Isotype Control (Sigma) (I5006)** | Rabbit | - |

Journal of Cell Science • Supplementary information

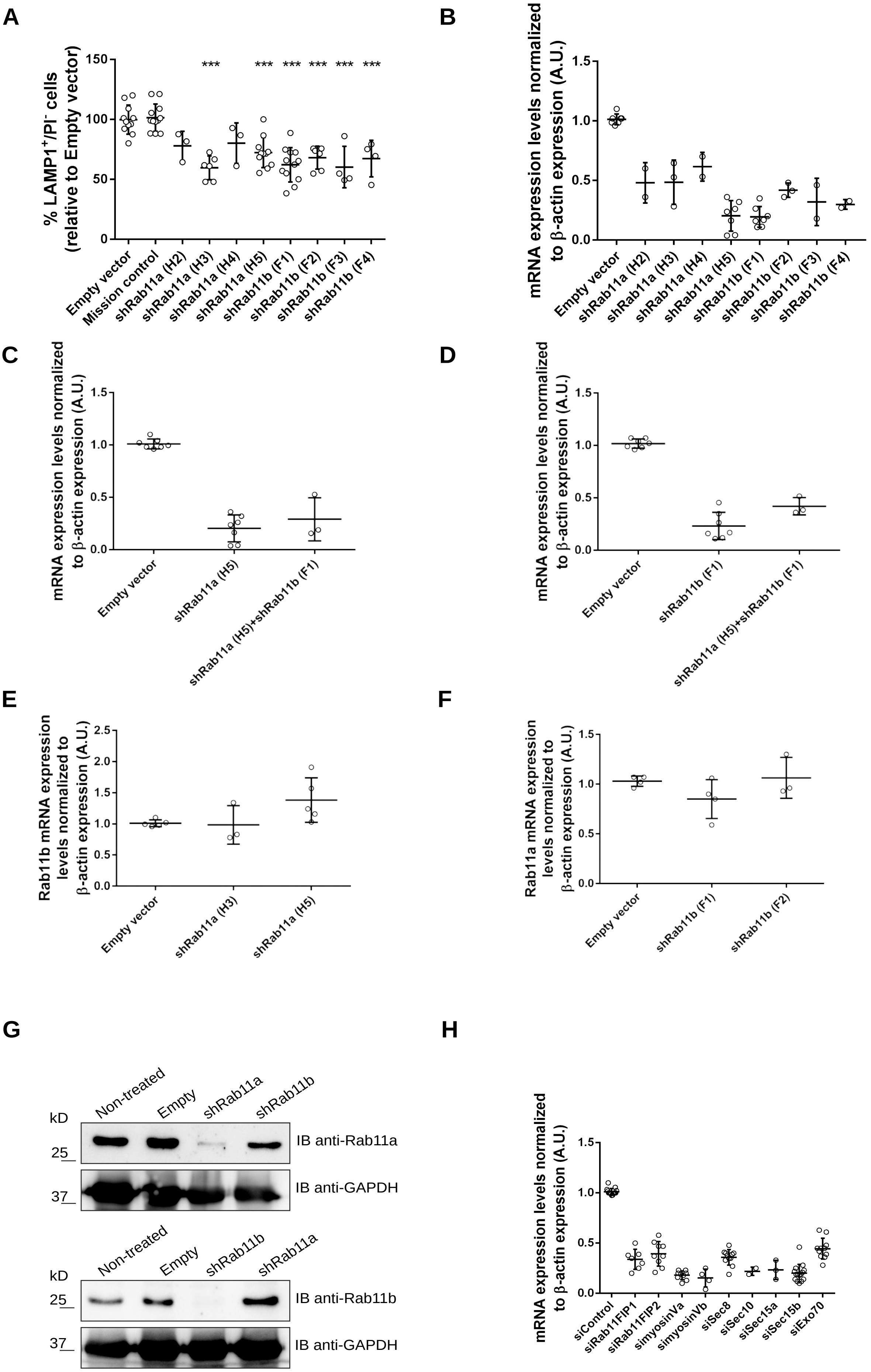
A
B
C
D
E
F
G
H
% LAMP1+/PI- cells (relative to Empty vector)
***
Empty vector
Mission control
shRab11a (H2)
shRab11a (H3)
shRab11a (H4)
shRab11a (H5)
shRab11b (F1)
shRab11b (F2)
shRab11b (F3)
shRab11b (F4)
mRNA expression levels normalized to β-actin expression (A.U.)
shRab11a (H5)+shRab11b (F1)
Rab11b mRNA expression levels normalized to β-actin expression (A.U.)
Rab11a mRNA expression levels normalized to β-actin expression (A.U.)
kD
Non-treated
Empty
shRab11a
shRab11b
IB anti-Rab11a
IB anti-GAPDH
IB anti-Rab11b
siControl
siRab11FIP1
siRab11FIP2
simyosinVa
simyosinVb
siSec8
siSec10
siSec15a
siSec15b
siExo70

**Fig. S1. Silencing efficiency of Rab11a, Rab11b and Rab11 effectors.** HeLa cells transduced with lentiviruses encoding different shRNAs targeting Rab11a or Rab11b were selected for 7 days with puromycin. (A) After treatment with 10 µM ionomycin and 4 mM $CaCl_2$ for 10 minutes at 37ºC, to trigger lysosome exocytosis, cells were collected, stained with anti-LAMP1 antibody and analyzed by flow cytometry. Plot represents the percentage of LAMP1-positive cells and PI-negative cells. Cells transduced with an empty vector (Empty) or a non-targeting shRNA (Mission) were used as negative controls. Results were normalized to the empty vector and are represented as mean ± SD of at least three independent experiments. ANOVA followed by Dunnett´s multiple comparisons test was used to compare different data sets with Empty vector (***$P<0.001$; all others are non-significant). (B) Rab11a and Rab11b silencing efficiency was assessed by qRT-PCR in HeLa cells transduced with lentiviruses encoding different shRNAs targeting Rab11a or Rab11b. Results were normalized for the expression of β-actin. (C) Rab11a expression was analyzed by qRT-PCR, as described before, when silenced alone or in combination with Rab11b. (D) Rab11b expression was analyzed by qRT-PCR, as described before, when silenced individually or in combination with Rab11a. (E) Rab11a expression was analyzed by qRT-PCR, as described before, when Rab11b was silenced with shRNAs targeting Rab11b. (F) Rab11b expression was analyzed by qRT-PCR, as described before, when Rab11a was silenced with shRNAs targeting Rab11a. All results were normalized to the empty vector and are represented as arbitrary units (A.U). Plots represent the mean ± SD of at least three independent experiments. (G) Rab11a and Rab11b protein expression levels were analyzed in HeLa cells transduced with lentiviruses encoding shRNA for Rab11a or Rab11b. Immunoblot was done using rabbit anti-Rab11a or anti-Rab11b antibodies. Non-transduced cells or cells transduced with empty vector were used as controls. Protein levels were normalized to GAPDH. The images are representative of three independent experiments. (H) Rab11 effectors were silenced in HeLa cells as described in Materials and Methods. The relative expression of each silenced gene was analyzed by qRT-PCR and normalized to the expression of β-actin. All results were normalized to siControl and are represented as arbitrary units (A.U). Results are representative of three independent experiments.

Journal of Cell Science • Supplementary information

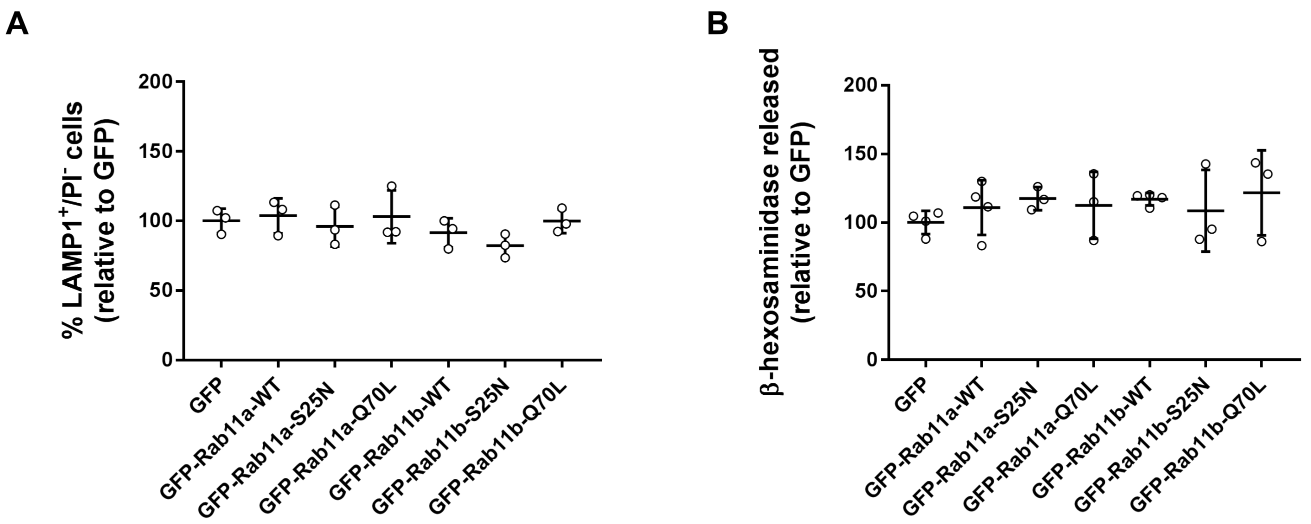


**Fig. S2. Overexpression of Rab11a or Rab11b wild-type or mutant forms does not affect LAMP1 cell surface translocation or β-hexosaminidase release.** (A) HeLa cells overexpressing the wild-type (WT), dominant negative (S25N) or constitutively active (Q70L) mutants of GFP-Rab11a or -Rab11b were treated with 10 μM ionomycin and 4 mM CaCl2 for 10 minutes at 37ºC, to trigger lysosome exocytosis. Cells were collected, stained with an anti-LAMP1 antibody and analyzed by flow cytometry. Plots represents the percentage of LAMP1-positive cells and PI-negative cells. (B) Cellular extracts and supernatants were collected and β-hexosaminidase release was quantified as described in Materials and Methods. Results were normalized to the cells transfected with a vector encoding GFP alone and are represented as mean ± SD of at least three independent experiments.

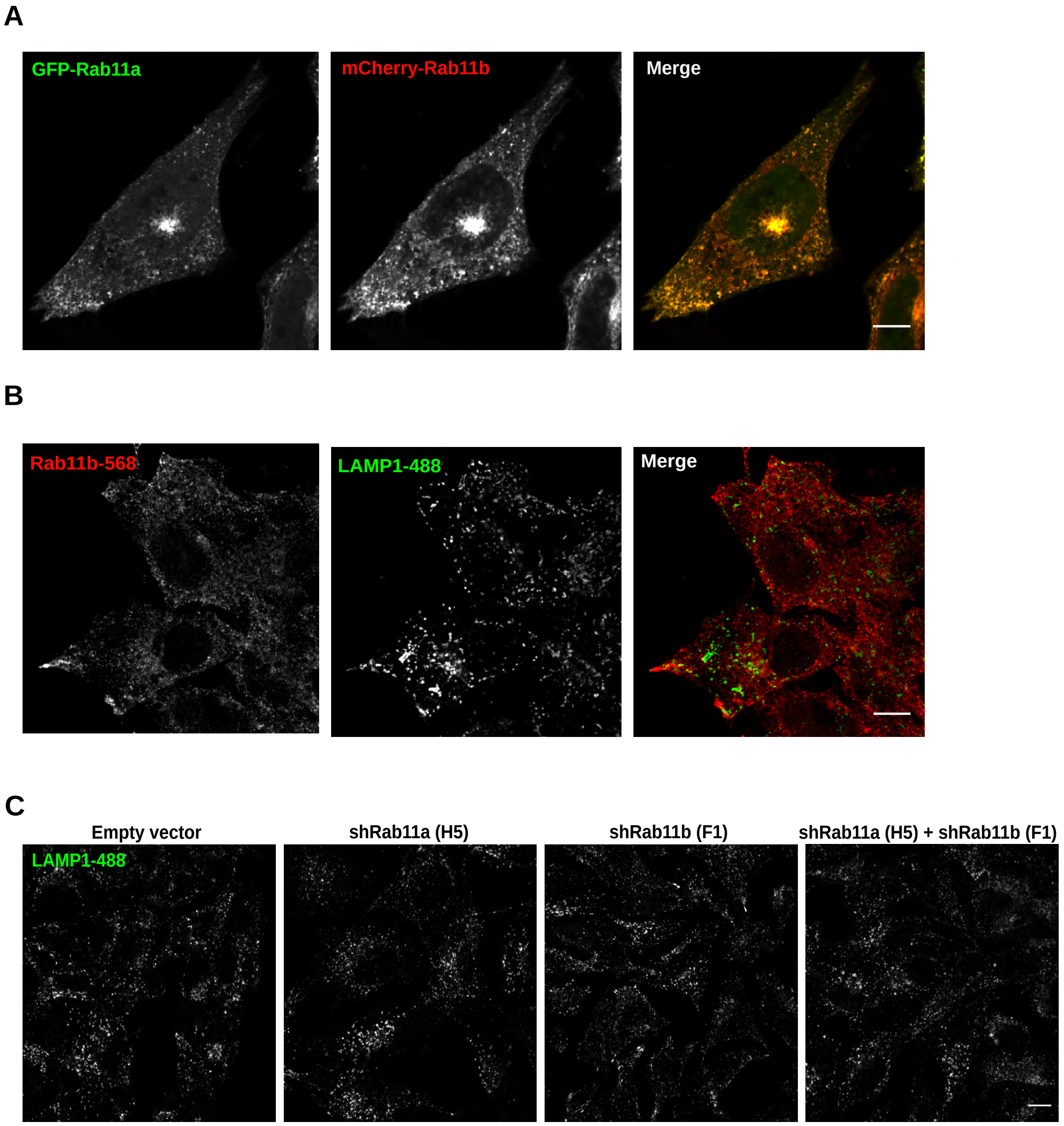


**Fig. S3. Rab11a and Rab11b display similar intracellular localization and LAMP1 distribution is not affected upon Rab11a or Rab11b silencing.** (A) Representative confocal microscopy images of the localization of Rab11a and Rab11b in HeLa cells transfected with both GFP-Rab11a (green) and mCherry-Rab11b (red) plasmids. Scale bar: 10 µm. (B) Confocal microscopy images of the intracellular localization of Rab11b and LAMP1, in HeLa cells, upon lysosome exocytosis stimulation with 2.5 µM ionomycin and 4 mM CaCl2. Cells were fixed and stained with rabbit anti-Rab11b (red) and mouse anti-LAMP1 (green) antibodies.  Scale bar: 10 µm. (C) Confocal microscopy images of the intracellular localization of LAMP1, in HeLa cells transduced with an empty vector (Empty) or lentiviruses encoding shRNAs targeting Rab11a (H5), Rab11b (F1) or both. Scale bar: 20 µm. Results are representative of three independent experiments where at least 15 cells were analyzed in each experiment.

Journal of Cell Science • Supplementary information

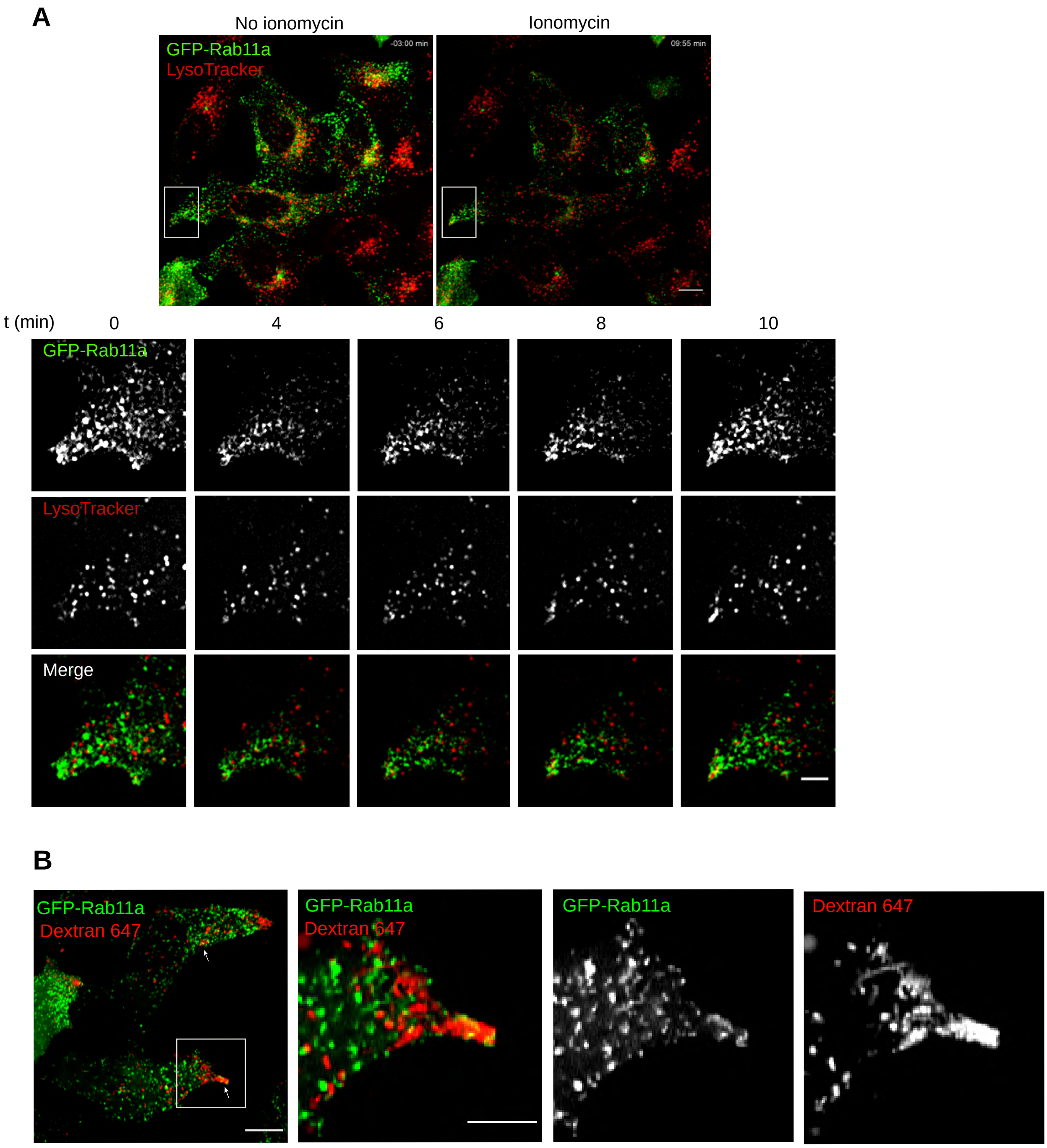


**Fig. S4. Colocalization of GFP-Rab11a with lysosomes labeled with dextran.** Live cell imaging of HeLa cells transiently transfected with GFP-Rab11a (green). (A) Cells incubated with LysoTracker (red) to label LEs/lysosomes were imaged in a spinning disk confocal microscope for 3 minutes before adding ionomycin and for 10 minutes after ionomycin stimulation. Images were captured every 5-10 seconds. Scale bar: 10 µm. The region outlined with a square was zoomed-in and the channels were split. Different time-frames: 0 (immediately after adding ionomyncin), 4, 6, 8 and 10 minutes are shown. Scale bar: 5 µm. Results are representative of two independent experiments, where at least 15 cells were analyzed in each experiment. (C) Cells were incubated overnight with dextran and chased for 4h. Cells were treated with ionomycin and imaged immediately. White arrows indicate the areas where dextran localize in close proximity with Rab11a-positive vesicles. Scale bar: 20 µm. The selected area was zoomed in. Scale bar: 10 µm. Results are representative of two independent experiments where at least 15 cells were analyzed in each experiment.

Journal of Cell Science • Supplementary information

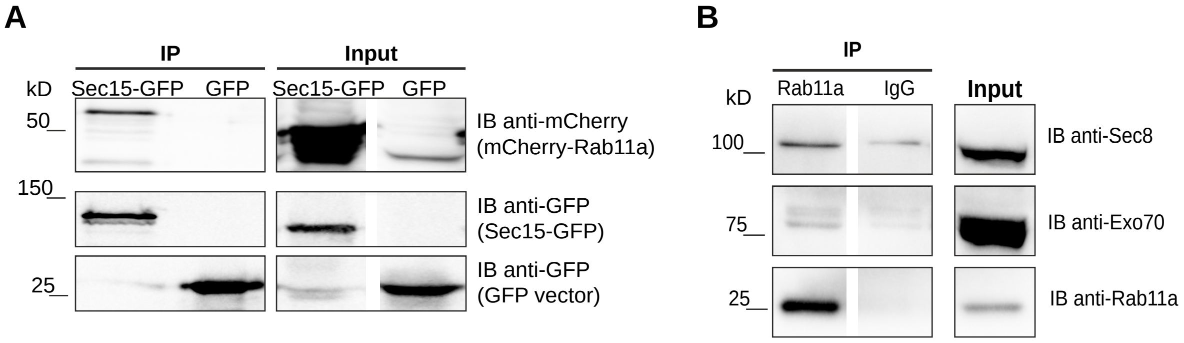


**Fig. S5. Rab11a co-immunoprecipitates with different exocyst complex subunits in HeLa cells.** (A) Total cell extracts (800 µg) were used to immunoprecipitate Sec15-GFP. Cells expressing GFP were used as a negative control. Immunoblot was done using goat anti-mCherry antibody to detect mCherry-Rab11a or goat anti-GFP antibody. (B) Total cell extracts (700 µg) were used to immunoprecipitate Rab11a, using rabbit anti-Rab11a antibody that recognizes specifically this isoform. Rabbit IgG was used as a negative control. Immunoblot was done using mouse anti-Sec8, mouse anti-Exo70 or rabbit anti-Rab11a antibodies. The images are representative of two or more independent experiments. Inputs correspond to 1/10 of total cell extracts used for immunoprecipitation. Results are representative of three independent experiments.

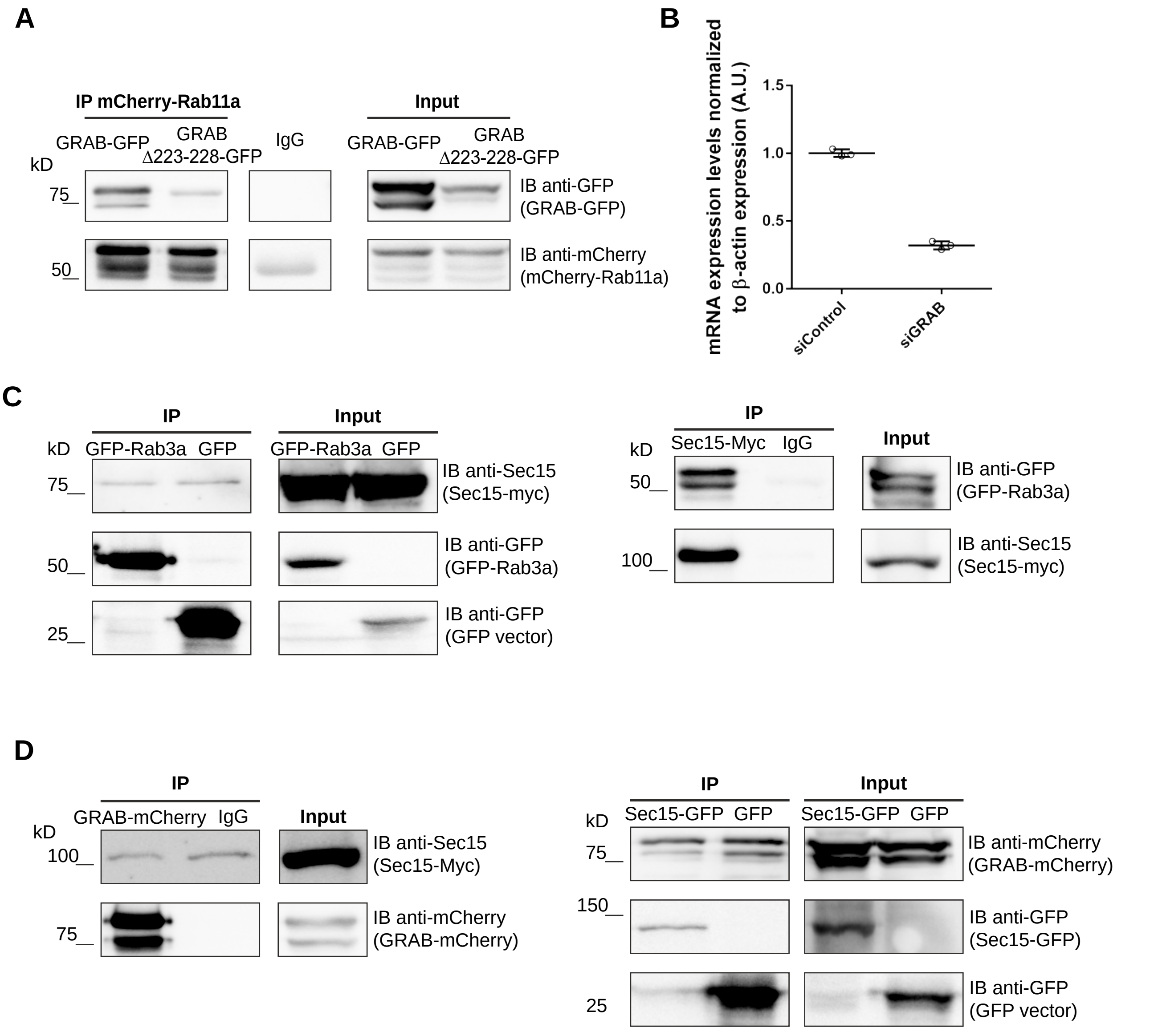


**Fig. S6. GRAB interacts with Rab11.** (A) Total cell extracts (600 µg) were used to immunoprecipitate mCherry-Rab11a using goat anti-mCherry antibody. Goat IgG was used as a negative control. Immunoblot was done using goat anti-GFP antibody to detect WT GRAB-GFP and GRAB Δ223-GFP. Input corresponds to 1/10 of total cell extracts used for immunoprecipitation. (B) GRAB was silenced in HeLa cells as described in Materials and Methods. Relative mRNA expression was analyzed by qRT-PCR and normalized to the expression of β-actin. Results are represented as arbitrary units (A.U). (C) Total cell extracts (350 µg) were used to immunoprecipitate GFP-Rab3a or Sec15-Myc, respectively. GFP-transfected cells or mouse IgG were used as negative controls. Immunoblot was done using goat anti-GFP or mouse anti-Sec15 antibodies to detect GFP-Rab3a or Sec15-Myc, respectively. Input corresponds to 1/10 of total cell extracts used for immunoprecipitation. (D) Total cell extracts (350-450 µg) were used to immunoprecipitate GRAB-mCherry or Sec15-GFP, respectively. GFP-transfected cells or goat IgG were used as negative controls. Immunoblot was done using goat anti-GFP, goat anti-mCherry or mouse anti-Sec15 antibodies to detect Sec15-GFP, GRAB-mCherry or Sec15-Myc, respectively. Input corresponds to 1/10 of total cell extracts used for immunoprecipitation. Results are representative of three independent experiments.

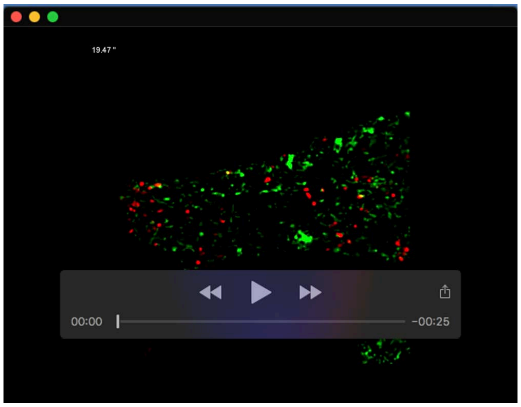


**Movie 1.** Live cell imaging of HeLa cells transiently transfected with GFP-Rab11a (green) and incubated for 1-2 hours with LysoTracker (red) to label LEs/ lysosomes. The cells were imaged in a super-resolution confocal microscope immediately after ionomycin stimulation. Images were captured every 6 seconds for 60 seconds. Results are representative of three independent experiments, where at least 8 cells were analyzed in each experiment.

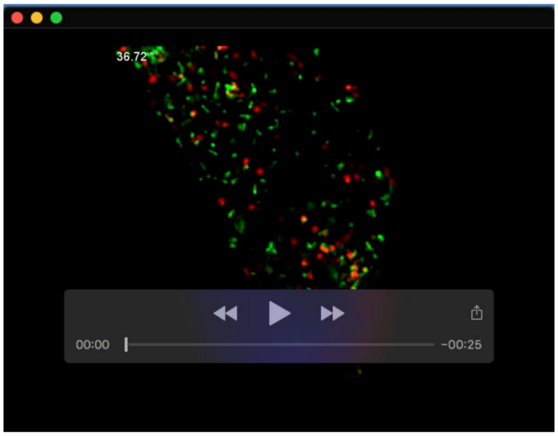


**Movie 2.** Live cell imaging of HeLa cells transiently transfected with GFP-Rab11a (green) and incubated for 1-2 hours with LysoTracker (red) to label LEs/lysosomes. The cells were imaged in a super-resolution confocal microscope immediately after ionomycin stimulation. Images were captured every 4 seconds for 90 seconds. Results are representative of three independent experiments, where at least 8 cells were analyzed in each experiment.

Journal of Cell Science • Supplementary information

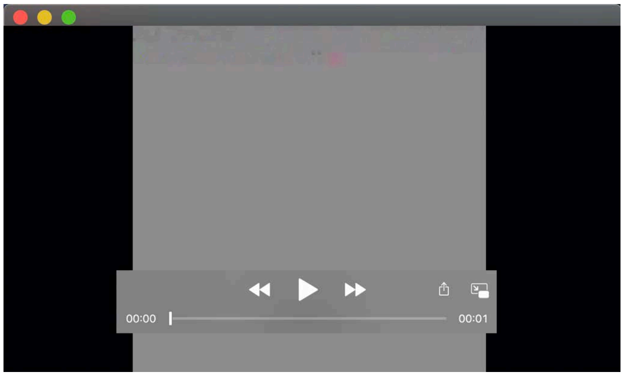


**Movie 3.** Live cell imaging of HeLa cells transiently transfected with GFP-Rab11a (green) and incubated for 1-2 hours with LysoTracker (red) to label lysosomes. The cells were imaged for 3 minutes before adding ionomycin (Time 0) and for 10 minutes after ionomycin stimulation. Images were captured every 5-10 seconds and are representative of two independent experiments, where at least 15 cells were analyzed in each experiment.

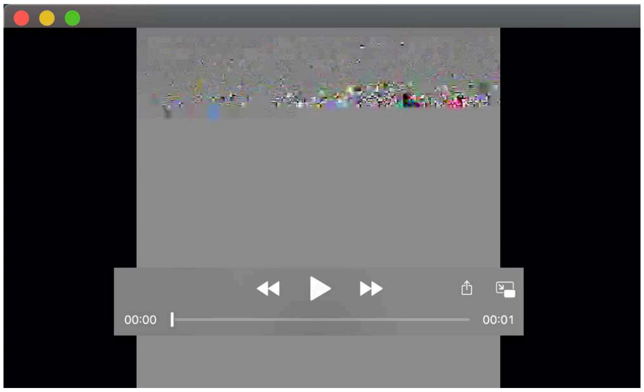


**Movie 4.** Live cell imaging of HeLa cells transiently transfected with GFP-Rab11b (green) and incubated for 1-2 hours with LysoTracker (red) to label lysosomes. The cells were imaged for 3 minutes before adding ionomycin (Time 0) and for 10 minutes after ionomycin stimulation. Images were captured every 5-10 seconds and are representative of two independent experiments, where at least 15 cells were analyzed in each experiment.

Journal of Cell Science • Supplementary information
