# Supplementary material for: Rab11 is required for lysosome exocytosis through the interaction with Rab3a, Sec15 and GRAB
Source: J Cell Sci. 2021 Jun 8;134(11):jcs246694. doi: 10.1242/jcs.246694 (PMC8214760; doi:10.1242/jcs.246694)
Supplement: Reviewer comments [file joces-134-246694_review_history.pdf]

Journal of Cell Science

Rab11 is required for lysosome exocytosis through the interaction with Rab3a, Sec15 and GRAB

Cristina Escrevente, Liliana Bento-Lopes, José S. Ramalho and Duarte C. Barral
DOI: 10.1242/jcs.246694

Editor: Mahak Sharma

Review timeline

Original submission:	15 April 2020
Editorial decision:	21 May 2020
First revision received:	22 September 2020
Editorial decision:	22 October 2020
Second revision received:	29 January 2021
Editorial decision:	3 March 2021
Third revision received:	22 April 2021
Accepted:	26 April 2021

Original submission

First decision letter

MS ID#: JOCES/2020/246694

MS TITLE: A novel Rab11-Rab3a cascade required for lysosome exocytosis

AUTHORS: Cristina Escrevente, Liliana Bento-Lopes, Jose S. Ramalho, and Duarte C. Barral
ARTICLE TYPE: Research Article

We have now reached a decision on the above manuscript.

To see the reviewers' reports and a copy of this decision letter, please go to: https://submit-jcs.biologists.org and click on the 'Manuscripts with Decisions' queue in the Author Area. (Corresponding author only has access to reviews.)

Both reviewers found the study to be interesting with new insights into regulation of lysosome exocytosis. As you will see, the reviewers also raise a number of substantial criticisms that prevent me from accepting the paper at this stage. They suggest, however, that a revised version might prove acceptable, if you can address their concerns. In particular, both reviewers have raised concern about the imaging data showing Rab11 association with LE/lysosomes. In addition, the reviewers have pointed out incomplete analyses, missing controls and better representation of graphical data.

If you think that you can deal satisfactorily with the criticisms on revision, I would be pleased to see a revised manuscript. We would then return it to the reviewers.

We are aware that you may currently be unable to access the lab to undertake experimental revisions. If it would be helpful, we encourage you to contact us to discuss your revision in greater detail. Please send us a point-by-point response indicating where you are able to address concerns raised (either experimentally or by changes to the text) and where you will not be able to do so within the normal timeframe of a revision. We will then provide further guidance. Please also note that we are happy to extend revision timeframes as necessary.

© 2021. Published by The Company of Biologists under the terms of the Creative Commons Attribution License (https://creativecommons.org/licenses/by/4.0/).

Please ensure that you clearly highlight all changes made in the revised manuscript. Please avoid using 'Tracked changes' in Word files as these are lost in PDF conversion.

I should be grateful if you would also provide a point-by-point response detailing how you have dealt with the points raised by the reviewers in the 'Response to Reviewers' box. Please attend to all of the reviewers' comments. If you do not agree with any of their criticisms or suggestions please explain clearly why this is so.

Reviewer 1

Advance summary and potential significance to field

This study examines the role of Rabs 11a and 11b and its interacting proteins in calcium-triggered exocytosis. While previous studies have found Rab11 to localize at post-Golgi vesicles and the recycling endosome, this study extends this RabÂ’s function to regulation of lysosome exocytosis. Rab11-labeled recycling endosomes at the cell periphery are suggested to transiently interact with lysosomes and affect their exocytosis through activation of Rab3a on the lysosomes through the GRAB protein. Rab11 interaction with Sec15 has also been implicated in this process, but the details of this remain scant. The study presents an interesting concept, but as discussed in my comments, technical limitations of the approaches and conjectural nature of many of the interpretation dampens the overall enthusiasm for this work.

Comments for the author

Major comments

1. siRNAs are known for their off-target effects, which has necessitated the approach of establishing the specificity of siRNA effects by re-expressing the siRNA-resistant cDNAs and examining if that rescues the shRNA knockdown effects at the functional level. This approach has not been utilized in case of any of the siRNAs used for this study. Not only should the authors provide rescue experiments, they should also present the data for their claimed 4 independent siRNAs used to silence Rab 11 expression, for which the data has not been presented.

2. The off-target effects of siRNAs are evident in case of the siRNA for Rab11b, which knocks down the expression of Rab11a protein (figure S1D - upper panel). In light of this, effect of over-expressing Rab11a protein in a Rab11b knockdown cell should be tested for its ability to reverse the effect on lysosome exocytosis. Moreover, for these assays GFP-tagged proteins could be used as this will help establish their functionality in the specific assays used here. Only upon such a validation can the inability of the overexpressed Rab11 (not Rab11-GFP) to enhance lysosome exocytosis in figure S2 can be established.

3. The claimed close localization of Rab11 and LAMP1 labeled vesicle is based on images from narrow and tightly-packed spaces at the tips of the cells, where there is an abundance of vesicles. This close apposition is lacking along the rest of the cell membrane. Unless the authors are implying that only lysosomes in these tiny tip regions of the cell undergo exocytosis, the claimed close apposition of the vesicles appears merely to be an artifact of crowding. As GRAB is the suggested bridge between the two vesicles, the effect of lacking GRAB binding to Rab3 or Rab11 on vesicle interactions at the tips should be examined and EN or super-resolution imaging should be used to truly resolve vesicles in this crowded environment.

4. Neither the videos 1, 2 nor the images shown in figure 2 offer any convincing evidence to support the claim that ionomycin triggers accumulation or selective loss of lysotracker-labeled vesicles at the cell’s periphery, undercutting the resulting claims.

5. Figure 3 makes a clear case that use of LAMP1 surface expression and b-hexosaminidase release can be significantly different following the same treatment, indicating that they report on rather different things. This, together with the authors indication that the b- hexosaminidase release is the true representation of lysosome exocytosis, while LAMP1 reports on LE as well, undercuts the claims made regarding lysosome exocytosis by using LAMP1 and lysotracker labeling.

© 2021. Published by The Company of Biologists under the terms of the Creative Commons Attribution License (https://creativecommons.org/licenses/by/4.0/).

6. Early in the study Rab11a and Rab11b knockdown has been shown to affect both LAMP1 and b-hexosaminidase release. However, later on upon finding that Sec15b knockdown (and not Sec15a knockdown) impacts on both of the above outcomes, the authors inexplicably switched to claiming that “Sec15a isoform seems to play a more specific role in lysosome exocytosis, while Sec15b may regulate both LE and lysosome exocytosis.” If the latter is the case then all of the earlier work with Rab 11 should also be discounted on this very basis.

7. Using the logic outlined above, the authors go on to examine localization and pull-down of Sec15a with Rab 11a, while ignoring to carry any such analysis of Sec15b. In view of the differences observed in the effects of Rab11a and Sec15a knockdowns vis-a-vie LAMP1 and b- hexosaminidase release, their co-localization and co-IP raises the question regarding the mechanism of differential effect of these knockdowns on LE/Ly exocytosis. Moreover, does Sec15b has the effect similar to Rab11? This should be examined using approaches presented for Sec15a.

8. The claimed weaker binding of mutant GRAB is based on the blot that shows the lower pull down of mutant goes hand in hand with lower starting input for this sample. Thus, this result in fact does not support the claim. Further, with the role of Rab3a in lysosome exocytosis, it is unclear how are authors establishing that the reduced b- hexosaminidase release in the GRAB knockdown cells is due to the effect on Rab 11-Rab3 interaction?

Minor comments

1. The opening section claims that Rab11a and Rab 11b were identified to be involved in regulation of lysosome exocytosis through a siRNA screen. However, no data or citation is offered for this and the studies cited there make no such claims. As this data forms the basis for this study, the authors should either provide such data or omit this claim. Moreover, as their previous screen did not identify Rab11 as a hit for lysosome exocytosis, the authors should discuss how their screen may have missed this and the impact of that on other potential Rabs that may regulate lysosome exocytosis and may have been missed in their previous study.

2. While Rab11 is found on biosynthetic and endocytic vesicles, the authors discuss Rab11 on the endocytic vesicles as the relevant pool. They should clarify if Rab11 on the post-Golgi vesicles could also contribute to the activation and if not what allows specificity for endocytic Rab11.

3. Instead of bar graphs, individual data should be presented (as dot plots) and the statistical tests should include the test of normal distribution of the data (and if so, this should be explicitly stated) before students t-test is applied to that data.

4. The term cell surface expression implies normal biosynthetic trafficking and localization of a protein, while it has been used here to imply cell surface translocation and should be corrected.

<u>Reviewer 2</u>

Advance summary and potential significance to field

In this study, the authors elucidate a role of Rab11a and b in Ca2+-induced lysosomal exocytosis, in cultured HeLa cells. They allude to a transient interaction of Rab11-positive with lysotracker positive vesicles at cell periphery (that is Ca2+ induced). Sec15, which interacts with Rab11 also appears to affect lysosome exocytosis, although other subunits of the exocyst complex do not appear to play a role. Lastly the Rab3 GEF, RAB also interacts with Rab11 (forming a potential molecular link between Rab11 and Rab3 positive vesicles). They thus propose an interesting model of a cascade where Rab11 recruits GRAB to cell periphery and this serves to recruit Rab3A for positioning of lysosomes for exocytosis(Rab3 having previously been implicated in this process). The topic of lysosome exocytosis is interesting given its role in many cellular process including membrane repair. The interaction of recycling compartments (rab11 positive vesicles) playing a role is also interesting and raises the possibility of organelle contacts being involved in this process as well

© 2021. Published by The Company of Biologists under the terms of the Creative Commons Attribution License (https://creativecommons.org/licenses/by/4.0/).

Comments for the author

While the FACS and hexosaminidase data is quite convincing (and thus implicates all the molecules mentioned above in the process), there are some issues that need to be addressed. The interaction of Rab11 with Sec 15, the involvement (or lack thereof, of other exocyst subunits) as well as how they envision Sec15 acting, should be better elucidated.

There are also details with regards to statistical analysis that need to be provided , as well as choice of images (in one particular case), used to convey colocalization that requires modification/better presentation.
These concerns need to be addressed prior to publication.
1. The images in Figure 2 are not convincing of colocalization: more zoomed in and higher resolution images of the still images of the time lapse need to be used, to convince one of the Rab11 and Lysotracker colocalization at the cell periphery(the zoomed in square appears pixelated and might require re-
processing). The colocalization of Sec 15 and Rab11 (Fig 4) is a good contrast where one can clearly appreciate the overlap.
2. Fig 4: the Sec15 interaction with Rab11 is relatively weak based on the IP blot; this should be mentioned in the text regarding the interaction.
3. The Checklist entered by the authors includes that individual data points need be displayed for experiments where n is less than 5; thus all the LAMP1 FACS experiments, hexosaminidase expts should display the data points in the graph, along with the mean of the 3 experiments currently shown. This is an important aspect of the journal's standard (and should be relatively straightforward for the authors to add using GraaphPad Prism that they have used).
4. While only Sec15 of exocyst complex appears to have an effect on lysosomal exocytosis, Rab 11 interacts with multiple subunits; the authors should explain this apparent contradiction. While Sec15 might well have an independent role in this process, is there a way to dissect this experimentally (other than siRNA effect alone)? Is it possible the depletion of the other subunits was less effective? Can visualizing the subunits with Rab11 be employed (under conditions of ionomycin treatment), to make this distinction?
5. The details of statistical analysis need to be more specific! The authors have indicated in the check list that cell #s in the different experiments have been mentioned, but this is often not (in fact almost never), the case. For instance, how many cells are examined for each representative confocal image, as in Fig.4C or for the time lapse videos? Please include this in methods or figure legends
6. The model proposed in the end indicates Sec15 interacts with Rab11 and possibly helps it move to the periphery but its interaction is then lost upon GRAB interaction. However, Fig4 clearly shows strong coloc of Rab11 and Sec15 in the cell periphery. How do the authors reconcile this data with their model? Time lapse videos examining Sec15 in this process with Rab11 might shed light on this. In fact visualizing Rab11 , Sec 15; Rab3 and Rab11 using live imaging might help strengthen the model proposed.
The study is on an interesting topic and highlights a new molecular cascade that plays a role in regulating lysosomal exocytosis. Addressing the concerns raised here will help strengthen their conclusion.

First revision

<u>Author response to reviewers' comments</u>

Reviewer 1

We thank the reviewer for acknowledging that our study "presents an interesting concept" and for critically analyzing it.

© 2021. Published by The Company of Biologists under the terms of the Creative Commons Attribution License (https://creativecommons.org/licenses/by/4.0/).

Major comments:

1. siRNAs are known for their off-target effects, which has necessitated the approach of establishing the specificity of siRNA effects by re-expressing the siRNA- resistant cDNAs and examining if that rescues the shRNA knockdown effects at the functional level. This approach has not been utilized in case of any of the siRNAs used for this study. Not only should the authors provide rescue experiments, they should also present the data for their claimed 4 independent siRNAs used to silence Rab 11 expression, for which the data has not been presented.

To confirm that Rab11a and Rab11b play a role in calcium-dependent lysosome exocytosis, we silenced Rab11a or Rab11b isoforms, in HeLa cells using five different shRNA hairpins, targeting different regions along the length of the transcript. For each isoform, we found four hairpins that efficiently silence Rab11a or Rab11b and show an impairment of LAMP1 cell surface translocation, upon ionomycin stimulation. This is now shown in the Supplementary Figure S1.

We proceeded our study with hairpins H3 and H5, for Rab11a and F1 and F2, for Rab11b because these efficiently silence Rab11a and Rab11b, respectively and do not affect the expression levels of the other Rab11 isoform transcript. Moreover Rab11a/b silencing with these hairpins induces a significant impairment in LAMP1 cell surface translocation and ß-hexosaminidase release, when compared with cells transduced with lentiviruses carrying an empty vector (Empty) or a vector encoding a non-targeting shRNA (Mission).

We agree with the Reviewer that rescue experiments are very important to establish the specificity of RNAi effects and we have performed them. For that, we transduced HeLa cells with shRNAs targeting Rab11a or Rab11b or an Empty vector. After seven days of selection, cells were transfected with GFP-Rab11a or GFP-Rab11b, respectively, or GFP. Calcium-induced lysosome exocytosis was then assessed, upon ionomycin stimulation, measuring LAMP1 cell surface translocation by flow cytometry and ß- hexosaminidase release. We observed that the overexpression of GFP-Rab11a or GFP- Rab11b in cells silenced for the same Rab11 isoform increases both LAMP1 cell surface translocation and ß-hexosaminidase release, as expected. However, we observed a similar increase in cells silenced for Rab11a or Rab11b and overexpressing GFP alone (see Fig. 1 below). Thus, cell transfection and protein overexpression by themselves induce lysosome secretion, possibly due to the stress caused to the cell by the uptake of the lipid/DNA complexes. Since the pool of lysosomes that undergo exocytosis is limited, ionomycin stimulation cannot provide any extra increase in lysosome exocytosis and therefore, the putative effect is masked.

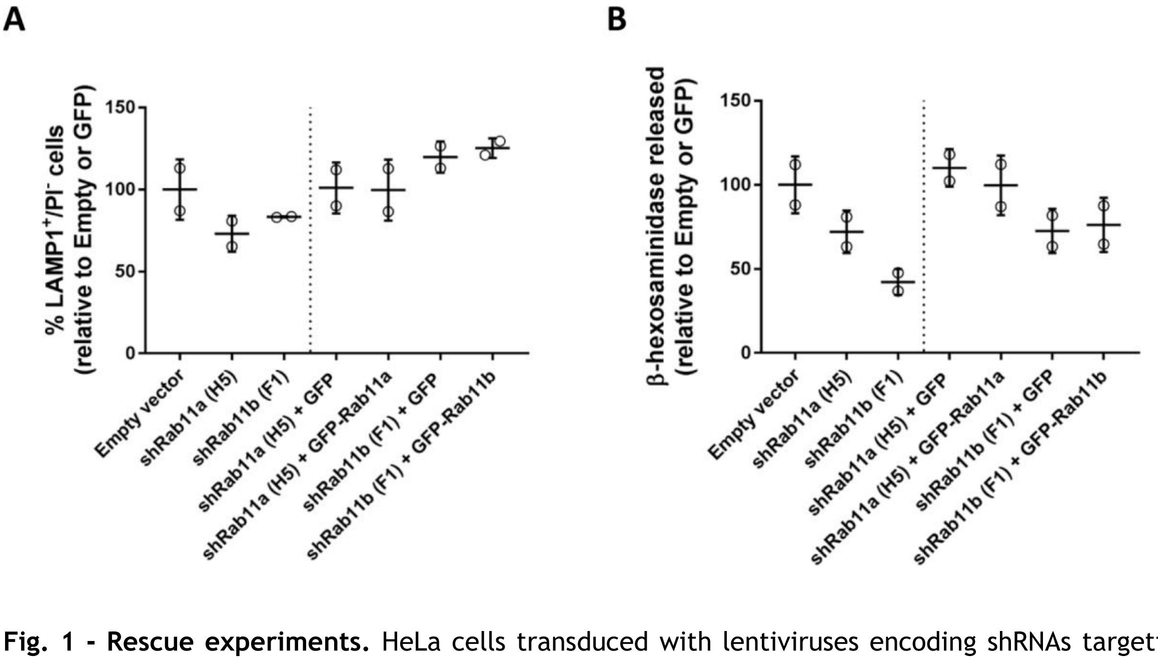

Fig. 1 - Rescue experiments. HeLa cells transduced with lentiviruses encoding shRNAs targeting

© 2021. Published by The Company of Biologists under the terms of the Creative Commons Attribution License (https://creativecommons.org/licenses/by/4.0/).

Rab11a or Rab11b were transfected with plasmids encoding GFP-Rab11a or GFP-Rab11b, respectively. As a control, transduced cells were transfected with a vector encoding GFP. Upon treatment with 10 µM ionomycin and 4 mM $CaCl_2$ for 10 minutes at 37°C, cells were collected, stained with anti-LAMP1 antibody and analyzed by flow cytometry. GFP-positive cells were gated and $LAMP1^+/PI^-$ cells analyzed are shown. Results were normalized to the empty vector and are represented as mean ± SD of two independent experiments.

Nevertheless, to show that the phenotype observed is not likely due to an off-target effect, we silenced Rab11a using siGENOME SMARTpool oligos in HeLa cells. As expected, we observed a reduction in LAMP1 cell surface translocation in Rab11a- silenced cells, upon ionomycin treatment, when compared with cells transfected with a non-targeting siRNA (siControl) (see Fig. 2 below).

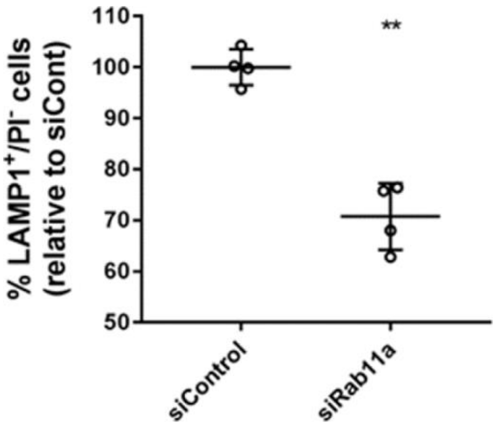

Fig. 2 - Rab11a silencing decreases LAMP1 cell surface translocation. HeLa cells silenced for Rab11a were treated with 10 µM ionomycin and 4 mM CaCl2 for 10 minutes at 37°C. Cells were collected, stained with an anti-LAMP1 antibody and analyzed by flow cytometry. Plot represents the percentage of $LAMP1^+/PI^-$ cells. Cells transfected with a non-targeting siRNA (siControl) were used as a negative control. Results were normalized to the siControl and are represented as mean ± SD of four independent experiments. Normal distribution of the data was determined using the Shapiro-Wilk normality test. Paired two-tailed Student's t-test was used to compare differences between siRab11a and siControl (**P<0.01).

2. The off-target effects of siRNAs are evident in case of the siRNA for Rab11b, which knocks down the expression of Rab11a protein (figure S1D - upper panel). In light of this, effect of over-expressing Rab11a protein in a Rab11b knockdown cell should be tested for its ability to reverse the effect on lysosome exocytosis. Moreover, for these assays GFP-tagged proteins could be used as this will help establish their functionality in the specific assays used here. Only upon such a validation can the inability of the overexpressed Rab11 (not Rab11-GFP) to enhance lysosome exocytosis in figure S2 can be established.

Although there is an apparent reduction in Rab11a protein expression upon Rab11b silencing, this decrease is also observed for the loading control (GAPDH). We further confirmed that that the silencing of Rab11b does not affect Rab11a expression levels, and vice-versa, by RT-PCR and this is now shown in the Supplementary Figure S1. Therefore, the silencing of Rab11a and Rab11b using the hairpins described is isoform- specific.

Concerning the reversion of the effect of Rab11b silencing on lysosome exocytosis by overexpressing Rab11a, it is an interesting possibility. However, as we explained in the previous point, cell transfection with a GFP-encoding vector increases lysosome exocytosis, precluding the conclusion as to whether the differences in lysosome exocytosis are due to the overexpressed protein.

3. The claimed close localization of Rab11 and LAMP1 labeled vesicle is based on images from narrow and tightly-packed spaces at the tips of the cells, where there is an abundance of vesicles. This close apposition is lacking along the rest of the cell membrane. Unless the

© 2021. Published by The Company of Biologists under the terms of the Creative Commons Attribution License (https://creativecommons.org/licenses/by/4.0/).

authors are implying that only lysosomes in these tiny tip regions of the cell undergo exocytosis, the claimed close apposition of the vesicles appears merely to be an artifact of crowding. As GRAB is the suggested bridge between the two vesicles, the effect of lacking GRAB binding to Rab3 or Rab11 on vesicle interactions at the tips should be examined and EN or super-resolution imaging should be used to truly resolve vesicles in this crowded environment.

Colocalization of Rab11-positive vesicles and late endosomes/lysosomes, labeled by LAMP1 is difficult to observe in fixed cells, most likely because the interaction is transient. Although we agree that the colocalization of Rab11-positive with LAMP1- positive late endosomes/lysosomes at the cell tips could be enhanced by the abundance of vesicles in a tightly-packed space, we now show by live cell imaging that Rab11a- positive vesicles colocalize transiently with late endosomes/lysosomes labeled with LysoTracker-red (new Supplementary Figure S4). Moreover, we can also observe colocalization of Rab11a-positive vesicles and lysosomes labeled with dextran (new Supplementary Figure S4).

We also agree that it would be interesting to see if in HeLa cells silenced for GRAB there are differences in the colocalization of Rab11-positive vesicles with late endosomes/lysosomes. Therefore, we performed confocal immunofluorescence studies in cells overexpressing GRAB to determine the colocalization with endogenous Rab11a or overexpressed Rab3a. However, due to the diffuse expression pattern displayed by GRAB, which localizes throughout the cytoplasm it was not possible to observe specific colocalization with Rab11a or Rab3a (see Fig. 3, below). Thus, we did not proceed with live cell imaging studies. Nevertheless, we postulate that GRAB is not essential for Rab11-positive vesicles to interact with late endosomes/lysosomes, *i.e.* in the absence of GRAB, Rab11-positive vesicles can still interact with Rab3a-positive late endosomes/lysosomes. However, in this condition Rab3a will not be activated, precluding lysosome positioning near the plasma membrane and consequently, exocytosis.

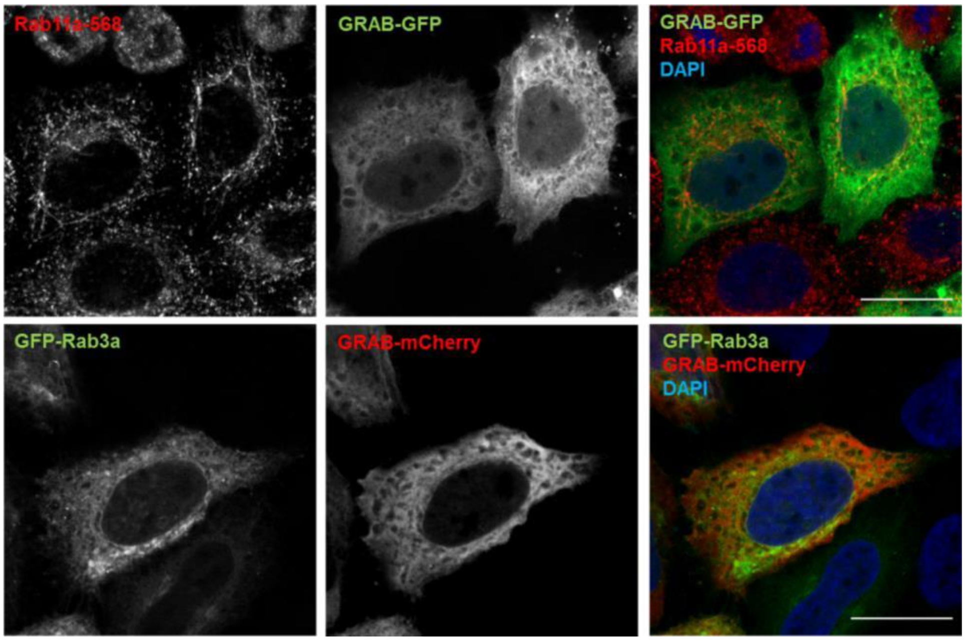

Fig. 3 - GRAB colocalization with Rab11a or Rab3a. Representative confocal microscopy images of the intracellular localization of overexpressed GRAB and endogenous Rab11a or overexpressed GFP-Rab3a. Cells were fixed and stained with rabbit anti-Rab11a antibody. Images were acquired on a Zeiss LSM 710 confocal microscope with a Plan-Apochromat 63x1.4 NA oil-immersion objective. Scale bar: 20 µm. Results are representative of two independent experiments.

4. Neither the videos 1, 2 nor the images shown in figure 2 offer any convincing evidence to support the claim that ionomycin triggers accumulation or selective loss of LysoTracker-labeled vesicles at the cell's periphery, undercutting the resulting claims.

© 2021. Published by The Company of Biologists under the terms of the Creative Commons Attribution License (https://creativecommons.org/licenses/by/4.0/).

Ionomycin stimulation leads to the exocytosis of a very small pool of lysosomes (around 10%, according to seminal studies from Norma Andrews group; *e.g.* Rodríguez, A., Webster, P., Ortego, J. and Andrews, N. W. (1997) Lysosomes behave as Ca^{2+}-regulated exocytic vesicles in fibroblasts and epithelial cells. *J. Cell Biol.* 137, 93–104). Moreover, these lysosomes localize close to the plasma membrane within seconds to few minutes.

We postulate that lysosomes that are close to the plasma membrane interact with Rab11/Rab3a-positive vesicles and then undergo exocytosis. Furthermore, lysosomes that are not at the cell periphery can also be recruited to the vicinity of the plasma membrane and undergo exocytosis. Indeed, we see an accumulation of LAMP1-positive and LysoTracker-positive vesicles at the cell periphery, upon ionomicyn stimulation, but this is not observed in all cells. Therefore, we removed this claim from the manuscript. Regarding the selective loss of LysoTracker-labeled vesicles, we do not expect it to be detectable with such small pool of lysosomes undergoing exocytosis and therefore, do not claim this.

5. Figure 3 makes a clear case that use of LAMP1 surface expression and b-hexosaminidase release can be significantly different following the same treatment, indicating that they report on rather different things. This, together with the authors indication that the b- hexosaminidase release is the true representation of lysosome exocytosis, while LAMP1 reports on LE as well, undercuts the claims made regarding lysosome exocytosis by using LAMP1 and lysotracker labeling.

LAMP1 surface expression reflects late endosome and lysosome exocytosis, while ß- hexosaminidase release is specific for lysosome exocytosis. Although LAMP1 and LysoTracker are not specific for lysosomes and also label late endosomes, a specific lysosome membrane marker is still to be found. One of the most reliable ways to mark lysosomes specifically is to incubate cells with dextran, wash and chase for a long period of time, to ensure that all internalized dextran accumulates in lysosomes. In fact, we tried this approach by live cell imaging and colocalized Rab11-positive vesicles with lysosomes labeled with dextran. Our results show that lysosomes labeled in this manner tend to be concentrated at the perinuclear region and almost do not move, when compared with late endosomes/lysosomes labeled with LysoTracker-red (see Fig. 4 below). Indeed, we have consistently observed that dextran-loaded lysosomes almost do not move, which is consistent with the loading slowing down transport. Nevertheless, we were able to observe colocalization between Rab11-positive vesicles and dextran- positive lysosomes by confocal live cell imaging, supporting our conclusions (new Supplementary Figure S4).

© 2021. Published by The Company of Biologists under the terms of the Creative Commons Attribution License (https://creativecommons.org/licenses/by/4.0/).

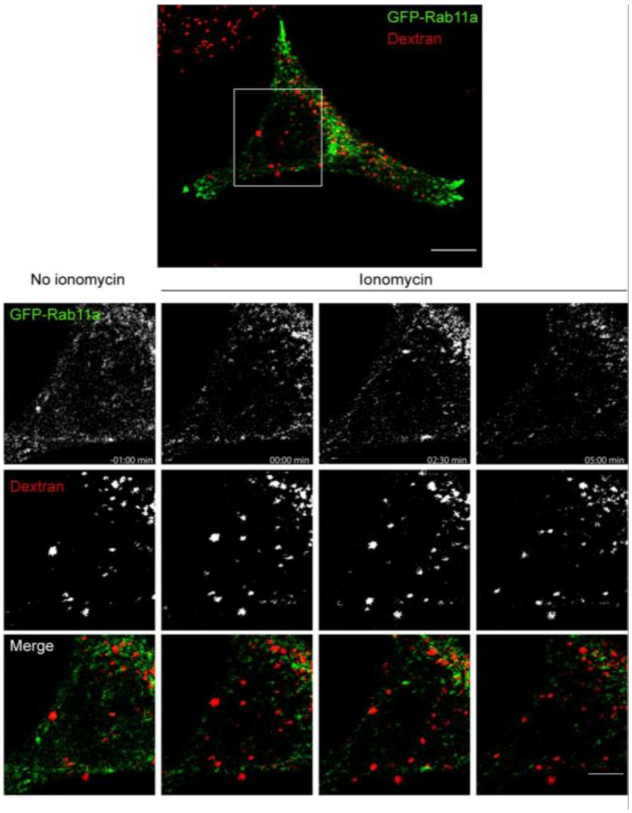

Fig. 4 - Colocalization between GFP-Rab11a and dextran-labeled lysosomes. Live cell imaging of HeLa cells transiently transfected with GFP-Rab11a (green), incubated overnight and chased for 2h with dextran-647 (red) to label lysosomes. The cells were imaged for 1 minute before adding ionomycin and for 5 minutes after ionomycin stimulation. Images were captured every 5 seconds. Scale bar: 10 µm. The region outlined with a square was zoomed-in and the channels were split. Different time-frames: no ionomycin (-01:00min) and with ionomycin stimulation (00:00; 02:30 and 5:00min) are shown. Scale bar: 5 µm. Images were acquired on a an Andor Revolution spinning disk confocal microscope equipped with an EMC CD camera with a Plan Apo VC PFS 60x 1.4 NA oil-immersion objective. Results represent two independent experiments and twenty cells analyzed.

6. Early in the study Rab11a and Rab11b knockdown has been shown to affect both LAMP1 and b-hexosaminidase release. However, later on upon finding that Sec15b knockdown (and not Sec15a knockdown) impacts on both of the above outcomes, the authors inexplicably switched to claiming that "Sec15a isoform seems to play a more specific role in lysosome exocytosis, while Sec15b may regulate both LE and lysosome exocytosis." If the latter is the case then all of the earlier work with Rab 11 should also be discounted on this very basis.

We provide evidence that Rab11a and Rab11b play a role in lysosome exocytosis but do not discard a role in late endosome exocytosis. We agree with the Reviewer that this possibility must be acknowledged and therefore, we now refer to it.

We suggest that Sec15a regulates lysosome exocytosis, while Sec15b could play a role in both late endosome and lysosome secretion, since Sec15b silencing impairs both ß- hexosaminidase release and the increase in LAMP1 surface expression, whereas Sec15a silencing only impairs ß-hexosaminidase release. Indeed, this could be the case if the main contribution to the increase in LAMP1 surface expression upon ionomycin treatment comes from late endosome exocytosis, which is not far-fetched if one considers that only about 10% of the lysosome pool undergoes exocytosis (see point 4). Of course, as the Reviewer refers, this would argue for a role of Rab11a/b also in late endosome exocytosis, which we have now acknowledged.

7. Using the logic outlined above, the authors go on to examine localization and pull-down of Sec15a with Rab 11a, while ignoring to carry any such analysis of Sec15b. In view of the differences observed in the effects of Rab11a and Sec15a knockdowns vis-a-vie LAMP1 and

© 2021. Published by The Company of Biologists under the terms of the Creative Commons Attribution License (https://creativecommons.org/licenses/by/4.0/).

b- hexosaminidase release, their co-localization and co-IP raises the question regarding the mechanism of differential effect of these knockdowns on LE/Ly exocytosis. Moreover, does Sec15b has the effect similar to Rab11? This should be examined using approaches presented for Sec15a.

Both Sec15a and Sec15b isoforms are presumed to interact with both Rab11a and Rab11b. However, while Sec15a has been extensively studied, close to nothing is known about Sec15b. To perform the reported experiments, we obtained a plasmid encoding rat Sec15a from Dr. Wei Guo and acquired a plasmid encoding rat Sec15b from genomics-online.com. Unfortunately, we found out that the latter plasmid only encodes half of the protein sequence. Importantly, the C-terminal region, which interacts with Rab11 is missing. Thus, we performed our studies with rat Sec15a only. Moreover, our aim was to confirm that in our model Rab11 co-immunoprecipitates with Sec15 and that Sec15 colocalizes with Rab11, in agreement with what has been described by others. Therefore, the use of Sec15a allowed us to fulfil this goal and while we agree with the Reviewer that it would be good to do a further characterization of Sec15b, this is beyond the scope of the study and would require the development of new tools.

8. The claimed weaker binding of mutant GRAB is based on the blot that shows the lower pull down of mutant goes hand in hand with lower starting input for this sample. Thus, this result in fact does not support the claim. Further, with the role of Rab3a in lysosome exocytosis, it is unclear how are authors establishing that the reduced b- hexosaminidase release in the GRAB knockdown cells is due to the effect on Rab 11-Rab3 interaction?

Indeed, the Reviewer makes a good point and we revisited this issue. We reanalyzed the western-blots, normalizing the IP band intensities for the respective inputs and controls.

Despite the difference pointed out by the Reviewer, the ratio between the intensity of the band corresponding to the IP and the input, in the Rab11a IP, normalized to the same ratio in the control (mCherry) IP is 0.5 for GRAB wt and 0.2 for the mutant GRAB. Therefore, it seems that the binding of the GRAB mutant to Rab11a is indeed weaker. Furthermore, we also show the reverse IP and in this case the inputs are similar, but there is still a weaker band for GRAB mutant, when compared to wt (Supplementary Figure S6). Thus, all the evidence supports the conclusion taken.

Rab3a in its active form plays an important role in the positioning and exocytosis of lysosomes, as shown by us before (Encarnação *et al.*, *J. Cell Biol.*, 2016). The fact that in the absence of GRAB, a known Rab3a GEF, we observe a decrease in lysosome exocytosis suggests that GRAB is important for Rab3a activation and the role of this Rab protein in lysosome exocytosis. Moreover, it was described by others and now by us that Rab11 co-immunoprecipitates with GRAB. In this study, we provide further evidence that Rab11 is required for late endosome/lysosome exocytosis. We also hypothesize that Rab11-positive vesicles can transport GRAB to the vicinity of Rab3a-positive lysosomes. In fact, we see that Rab11 and Rab3a co-immunoprecipitate. Thus, we postulate that a Rab11-GRAB-Rab3a cascade exists and is required for lysosome exocytosis.

Minor comments

1. The opening section claims that Rab11a and Rab 11b were identified to be involved in regulation of lysosome exocytosis through a siRNA screen. However, no data or citation is offered for this and the studies cited there make no such claims. As this data forms the basis for this study, the authors should either provide such data or omit this claim. Moreover, as their previous screen did not identify Rab11 as a hit for lysosome exocytosis, the authors should discuss how their screen may have missed this and the impact of that on other potential Rabs that may regulate lysosome exocytosis and may have been missed in their previous study.

The shRNA screen reported in Encarnação *et al.* (*J. Cell Biol.*, 2016) was performed in THP-1 cells, to identify Rab GTPases involved in lysosome exocytosis and plasma membrane repair. The screen included 58 Rabs with four to five different shRNAs for each Rab (detailed in Table S1 of the referred publication). Moreover, it resulted in the identification of several Rab proteins that,

© 2021. Published by The Company of Biologists under the terms of the Creative Commons Attribution License (https://creativecommons.org/licenses/by/4.0/).

when silenced reduce LAMP1 levels at the plasma membrane, upon ionomycin stimulation. For a Rab protein to be considered as a positive hit, at least three shRNAs had to result in impaired LAMP1 cell surface translocation. Among the hits were Rab3a, Rab10 and also Rab11b. In Fig. S1A of the published study by Encarnação *et al.*, a dot plot shows the results of the screen, identifying Rab3a and Rab10 as hits. Below, we show a plot of the same data with Rab11b identified (Fig. 5). Despite Rab11b not being explicitly referred in the published work by Encarnação *et al.*, the data where we base our claim was generated within the published study. For this reason, we left the reference to Encarnação *et al.*

[Figure provided for reviewer has been removed. It was based on data published in **Encarnação**, Marisa et al. "A Rab3a-dependent complex essential for lysosome positioning and plasma membrane repair." *The Journal of cell biology* vol. **213**,6 (2016): 631-40. doi:10.1083/jcb.201511093.]

2. While Rab11 is found on biosynthetic and endocytic vesicles, the authors discuss Rab11 on the endocytic vesicles as the relevant pool. They should clarify if Rab11 on the post-Golgi vesicles could also contribute to the activation and if not what allows specificity for endocytic Rab11.

Throughout the manuscript, we always refer to Rab11-positive vesicles. Nevertheless, we now refer that Rab11 was also described to regulate the secretory pathway and in the model we refer that the Rab11-positive vesicles could be derived from the endocytic recycling compartment or the Golgi.

3. Instead of bar graphs, individual data should be presented (as dot plots) and the statistical tests should include the test of normal distribution of the data (and if so, this should be explicitly stated) before students t-test is applied to that data.

We followed the Reviewer's suggestion and changed the graphs to dot plots. Individual data distribution and the number of experiments are now clearly visible in all assays. Regarding the statistical analysis, our data follows a normal distribution and passed the Shapiro-Wilk normality test. This is now stated in the description of the statistical analysis in the Materials and Methods section. Moreover, we applied the One-way ANOVA followed by Dunnett's multiple comparisons test, since we recognize that it is a more robust method and the statistical significance is maintained, except in the case of MyosinV and ß-hexosaminidase release. In Fig. 6B, we only have two groups and therefore, we used a paired t-test, since ANOVA cannot be used in this situation.

4. The term cell surface expression implies normal biosynthetic trafficking and localization of a protein, while it has been used here to imply cell surface translocation and should be corrected.

The term "cell surface expression" was corrected to "cell surface translocation" in the manuscript, following the Reviewer's suggestion.

We would like to thank the Reviewer for the insightful comments and suggestions for improvement.

Reviewer 2

We thank the Reviewer for acknowledging that in our study we "propose an interesting model" and for the insightful comments.

1. The images in Figure 2 are not convincing of colocalization: more zoomed in and higher resolution images of the still images of the time lapse need to be used, to convince one of the Rab11 and Lysotracker colocalization at the cell periphery(the zoomed in square appears pixelated and might require re-processing). The colocalization of Sec 15 and Rab11 (Fig 4) is a good contrast where one can clearly appreciate the overlap.

Colocalization between Rab11 and LAMP1 in fixed cells is not easy to demonstrate, similar to what was reported for the colocalization between Rab3a and LAMP1 (Encarnação *et al.*, *J. Cell Biol.*, 2016). This is likely due to the transient nature of the interaction between Rab11-positive vesicles

© 2021. Published by The Company of Biologists under the terms of the Creative Commons Attribution License (https://creativecommons.org/licenses/by/4.0/).

and late endosomes/lysosomes. Nevertheless, in confocal time-lapse live cell microscopy, we clearly observed Rab11- positive vesicles interacting with late endosomes/lysosomes labeled with LysoTracker (Supplementary Figure S4). To better show the colocalization, we improved the images obtained by re-processing the zoom-ins and the results are now clearer (see Figure 2). Moreover, we used a Zeiss LSM 980 confocal microscope with Airyscan to acquire time- lapse images of live cells. The results confirm the colocalization between GFP-Rab11a and LysoTracker with super resolution (see new Figure 2C).

2. Fig 4: the Sec15 interaction with Rab11 is relatively weak based on the IP blot; this should be mentioned in the text regarding the interaction.

In response to this comment, we introduced the following sentence in the text: “Noteworthy, albeit weakly, we observed that GFP-Rab11 co-immunoprecipitates with myc-tagged Sec15a…”.

3. The Checklist entered by the authors includes that individual data points need be displayed for experiments where n is less than 5; thus all the LAMP1 FACS experiments, hexosaminidase expts should display the data points in the graph, along with the mean of the 3 experiments currently shown. This is an important aspect of the journal’s standard (and should be relatively straightforward for the authors to add, using GraaphPad Prism that they have used).

We followed the Reviewer’s advice and the bar graphs were replaced by dot plots. Individual data distribution, means and the number of experiments are now clearly represented in all graphs.

4. While only Sec15 of exocyst complex appears to have an effect on lysosomal exocytosis, Rab 11 interacts with multiple subunits; the authors should explain this apparent contradiction. While Sec15 might well have an independent role in this process, is there a way to dissect this experimentally (other than siRNA effect alone)? Is it possible the depletion of the other subunits was less effective? Can visualizing the subunits with Rab11 be employed (under conditions of ionomycin treatment), to make this distinction?

Few studies have addressed the possible role of the exocyst subunits independently of the exocyst complex and it remains elusive if there are functional subcomplexes. For example, Mehta *et al.* suggest that different exocyst components or subcomplexes have separable functions, in *Drosophila* (Mehta *et al., Neuron,* 2005). Moreover, the authors observed that the function of other exocyst components are maintained in the absence of Sec15. Furthermore, Sec15 is described to interact directly with Rab11, while the other exocyst subunits interact with Rab11 when the exocyst complex is assembled (Wu *et al., Nat. Struct. Mol. Biol.*, 2005; Zhang *et al., J. Biol. Chem.*, 2004).

The Reviewer is right that the silencing efficiency of Sec15 is higher than the other subunits, but not by more than 20%. To shed light on this issue, we followed the Reviewer’s suggestion and performed confocal immunofluorescence microscopy studies. The results show striking colocalization between GFP-Sec15 and endogenous Rab11a in the absence or presence of ionomycin (see Figure 4C and Fig. 6, below), while no colocalization is observed between endogenous Rab11a and GFP-Exo70, GFP-Sec8 or mCherry-Sec10. GFP-Exo70 is mostly expressed at the plasma membrane in non-treated cells. Upon ionomycin treatment, GFP-Exo70 localization becomes more diffuse throughout the cytoplasm but no specific colocalization is observed with Rab11a. GFP- Sec8 and mCherry-Sec10 show a diffuse pattern in the cytoplasm with no colocalization with endogenous Rab11a either in the absence or presence of ionomycin. Thus, the colocalization data reinforces the conclusion that Rab11 interacts with Sec15 to regulate lysosome secretion. In conclusion, although our results point to a role of Sec15 independently of the exocyst complex, we cannot exclude that other exocyst subunits or subcomplexes are also involved in lysosome exocytosis, since we did not silence all subunits. Therefore, we have referred this in the Discussion.

Furthermore, we now do not discuss the existence of a Rab11-Sec15-MyosinVa complex being required for lysosome exocytosis, since we do not have enough data to support this claim.

© 2021. Published by The Company of Biologists under the terms of the Creative Commons Attribution License (https://creativecommons.org/licenses/by/4.0/).

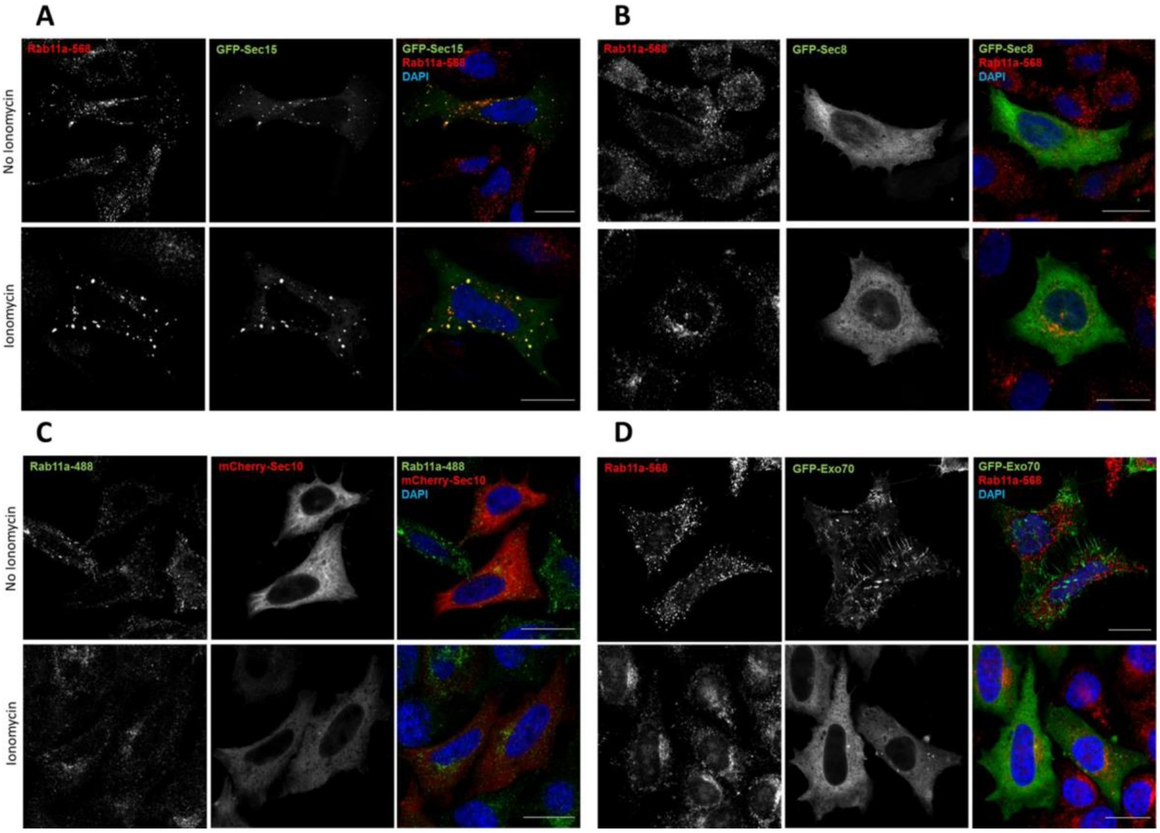

Fig. 6 - Colocalization between Rab11a and GFP-tagged exocyst subunits. Representative confocal microscopy images of HeLa cells overexpressing the exocyst subunits GFP-Sec15a (A), GFP-Sec8 (B), mCherry-Sec10 (C) or GFP-Exo70 (D) and stained with rabbit anti-Rab11a antibody. Cells were analyzed in the absence or presence of 10 µM ionomycin and 4 mM CaCl2 for 10 minutes at 37°C, to trigger lysosome exocytosis. Images were acquired on a Zeiss LSM 710 confocal microscope with a Plan-Apochromat 63x1.4 NA oil-immersion objective. Scale bar: 20 µm. Results are representative of two independent experiments.

5. The details of statistical analysis need to be more specific! The authors have indicated in the check list that cell #s in the different experiments have been mentioned, but this is often not (in fact almost never), the case. For instance, how many cells are examined for each representative confocal image, as in Fig.4C or for the time lapse videos? Please include this in methods or figure legends

Following the Reviewer's advice, the information regarding the number of experiments and cells analyzed was added to the Materials and Methods section and also to the figure legends.

6. The model proposed in the end indicates Sec15 interacts with Rab11 and possibly helps it move to the periphery but its interaction is then lost upon GRAB interaction. However, Fig4 clearly shows strong coloc of Rab11 and Sec15 in the cell periphery. How do the authors reconcile this data with their model? Time lapse videos examining Sec15 in this process with Rab11 might shed light on this. In fact visualizing Rab11 , Sec 15; Rab3 and Rab11 using live imaging might help strengthen the model proposed.

It is true that GFP-Sec15 colocalizes with Rab11 at the cell periphery and close to the plasma membrane, but also in other areas of the cell. This colocalization throughout the cell occurs both with and without ionomycin stimulation, as depicted in the Figure below (Fig. 7). Although we did not observe co-immunoprecipitation of GFP-Rab3a or GRAB- GFP with myc-Sec15a (Fig. S6C, D), suggesting that Rab11 interaction with Sec15 could be lost before Rab11 interacts with GRAB and Rab3a, we cannot exclude that Sec15 interaction with Rab11 also occurs/remains at the periphery. Therefore, we only refer to the former as a possibility ("…since we could not detect an obvious interaction between GRAB or Rab3a and Sec15, it is possible that Rab11 interacts with

© 2021. Published by The Company of Biologists under the terms of the Creative Commons Attribution License (https://creativecommons.org/licenses/by/4.0/).

GRAB and Rab3a after interacting with Sec15…").

We also attempted to colocalize GFP-Rab3a and mCherry-Rab11a, but when Rab3a is co-overexpressed with Rab11a it shows a diffuse distribution that precludes firm conclusions about their colocalization (see Fig. 8, below).

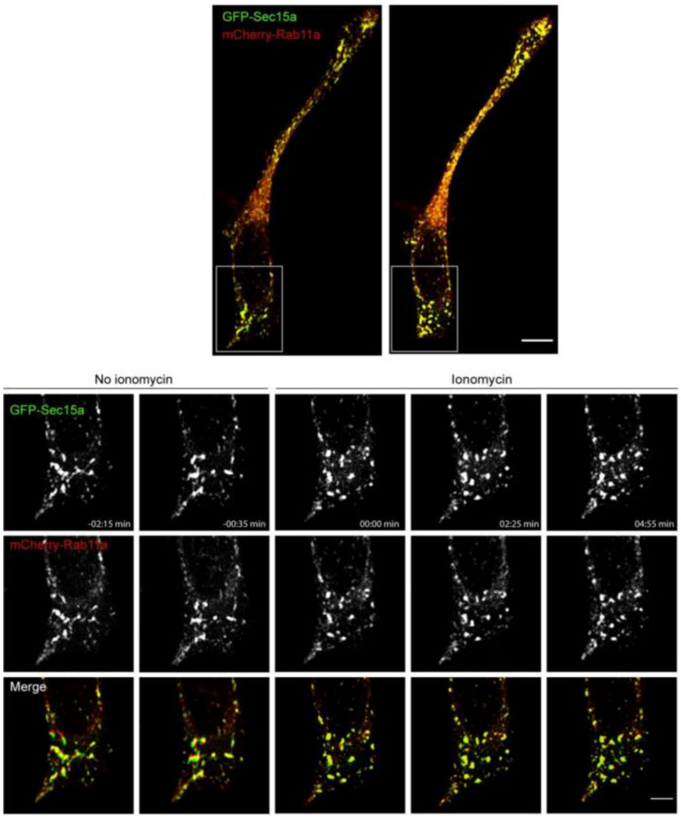

Fig. 7 - Colocalization between mCherry-Rab11a and GFP-Sec15a. Live cell imaging of HeLa cells transiently transfected with mCherry-Rab11a (red) and GFP-Sec15 (green). The cells were imaged for 3 minutes before adding ionomycin and for 8 minutes after ionomycin stimulation. Images were captured every 5 seconds. Scale bar: 10 µm. The region outlined with a square was zoomed-in and the channels split. Several time-frames are shown: no ionomycin (-02:15 and –00:35 min) and with ionomycin stimulation (00:00; 02:25 and 4:55 min). Scale bar: 5 µm. Images were acquired on a an Andor Revolution spinning disk confocal microscope equipped with an EMC CD camera with a Plan Apo VC PFS 60x 1.4 NA oil-immersion objective. Results are representative of ten cells analyzed.

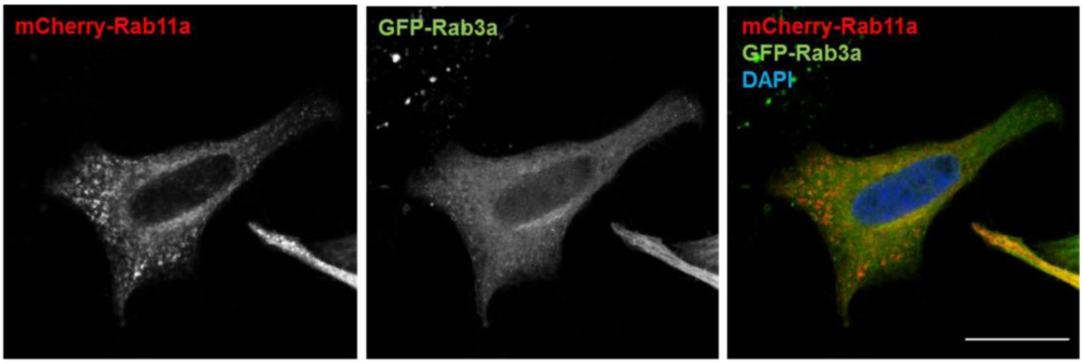

Fig. 8 - Colocalization between mCherry-Rab11a and GFP-Rab3a. Representative confocal

© 2021. Published by The Company of Biologists under the terms of the Creative Commons Attribution License (https://creativecommons.org/licenses/by/4.0/).

microscopy images of the intracellular localization of overexpressed mCherry-Rab11a and GFP-Rab3a in fixed cells. Images were acquired on a Zeiss LSM 710 confocal microscope with a Plan-Apochromat 63x1.4 NA oil- immersion objective. Scale bar: 20 µm. Results are representative of two independent experiments.

We would like to thank the Reviewer for the careful analysis of the manuscript and suggestions for improving it.

Second decision letter

MS ID#: JOCES/2020/246694

MS TITLE: A novel Rab11-Rab3a cascade required for lysosome exocytosis

AUTHORS: Cristina Escrevente, Liliana Bento-Lopes, Jose S. Ramalho, and Duarte C. Barral
ARTICLE TYPE: Research Article

We have now reached a decision on the above manuscript.

To see the reviewers' reports and a copy of this decision letter, please go to: https://submit-jcs.biologists.org and click on the 'Manuscripts with Decisions' queue in the Author Area. (Corresponding author only has access to reviews.)

As you will see, both reviewers think that the manuscript is improved after revision, however, reviewer 1 still finds substantial major concerns with the revised version that prevent me from accepting the paper at this stage. As reviewer 1 has suggested and I also agree, that a revised version might prove acceptable, if you can address these concerns. If you think that you can deal satisfactorily with the criticisms on revision, I would be pleased to see a revised manuscript. We would then return it to the reviewer 1.

In particular, there are still two major concerns that are not satisfactorily answered by the evidence presented in the manuscript. First, the manuscript lacks clear evidence of the claim that â€œRab11-positive vesicles transiently interact with lysosomes at the cell peripheryâ€•. None of the imaging data shown resolves the concern that Rab11 vesicles and lysosomes are simply closely packed in the cell periphery. Even if these vesicles are interacting transiently, better imaging/resolution is required to prove this conclusion. Probably a technique like proximity ligation assay can be used to show transient interaction between different proteins on membranes.

A second major concern is how does Rab11 mediate lysosome exocytosis? Figure 7 suggests an interesting working model. However, the current version of the manuscript lacks experimental evidence to support such a model. For instance, does Rab11-GRAB and Rab3 form a complex and whether Rab11 depletion changes Rab3 association with lysosomes? Also, whether the constitutively active form of Rab3 overcome lysosome exocytosis defect upon Rab11 depletion.

The concern raised by reviewer 1 on the lack of a rescue in the shRNA experiments is also relevant to the major conclusion of the manuscript. Probably, reintroduction of Rab11 rescue construct using low-copy plasmids or inducible plasmids might be helpful.

We are aware that you may be experiencing disruption to the normal running of your lab that makes experimental revisions challenging. If it would be helpful, we encourage you to contact us to discuss your revision in greater detail. Please send us a point-by-point response indicating where you are able to address concerns raised (either experimentally or by changes to the text) and where you will not be able to do so within the normal timeframe of a revision. We will then provide further guidance.

© 2021. Published by The Company of Biologists under the terms of the Creative Commons Attribution License (https://creativecommons.org/licenses/by/4.0/).

Please ensure that you clearly highlight all changes made in the revised manuscript. Please avoid using 'Tracked changes' in Word files as these are lost in PDF conversion.

I should be grateful if you would also provide a point-by-point response detailing how you have dealt with the points raised by the reviewers in the 'Response to Reviewers' box. Please attend to all of the reviewers' comments. If you do not agree with any of their criticisms or suggestions please explain clearly why this is so.

Reviewer 1

Advance summary and potential significance to field

The revised manuscript has explained some of the findings that needed clarification, which has improved parts of the manuscript identifying the role of Rab 11 in the exocytic fate of late endosomes / lysosomes.

Comments for the author

Additional work is needed to address all of the previous comments and some of the responses are source of confusion or contradictory statements. These are all discussed below.

• Use of a pool of siGENOME siRNAs does little to establish the specificity of shRNA-mediated knockdown and its rescue by re-expressing the shRNA resistant GFP-Rab11a/b. Assuming shRNA resistant GFP-Rab11a/b cDNAs were used for the rescue (were they?), it is evident that these plasmids offer similar level of rescue of LAMP1 and b-hex secretion as GFP only cDNA. To explain this discrepancy, authors claim that "…protein overexpression by themselves induce lysosome secretion, possibly due to the stress caused to the cell by the uptake of the lipid/DNA complexes.". If transfections do indeed alter lysosomal behavior in the experimental conditions used here, then this raises grave concerns regarding the validity of experiments performed throughout the manuscript (e.g. Figures 2, 4, 5, 6, and several supplemental data) that uses transfection to express fluorescently-tagged Rabs. This issue needs to be clearly resolved.

• To address if the perceived colocalization of Rab11 and LAMP1-positive vesicles at the cell tips is not due to the vesicles being tightly packed vesicles in a that space, authors carried out live cell imaging of Rab11a with LysoTracker/Dextran 647. While there is no lysotracker data in figure S4, the data shown with dextran 647 again fails to resolve individual dextran-labeled vesicle. So, this data does little to answer the original concern regarding need for greater resolution by using electron microscopy (EM) of super-resolution microscopy of the vesicles at the cell tip. The only effort in this direction has been presented in revised figure 2C showing vesicles adjacent to the perinuclear region where there is no vesicle crowding and no expectation for co-localization as stated that Rab11-positive vesicles "…interact with LEs/lysosomes at the cell periphery just before lysosome fusion with the plasma membrane." Thus, this co-localization in the perinuclear space is puzzling and a clear evidence for co-localization at cell tip is still lacking.

• In the manuscript text authors state that "…we postulated that Rab11 and Rab3a interaction is mediated by an adaptor protein. [GRAB]". However, in their response authors contradict themselves by speculating "we postulate that GRAB is not essential for Rab11-positive vesicles to interact with late endosomes/lysosomes, i.e. in the absence of GRAB, Rab11-positive vesicles can still interact with Rab3a-positive late endosomes/lysosomes." This contradiction needs to be resolved as this data is critical to the overall model proposed by this study.

• In another contradiction, in the manuscript authors make the claim "Upon ionomycin stimulation, the increase in intracellular calcium concentration leads to colocalization of LysoTracker-positive vesicles with GFP Rab11a-positive vesicles, for a few seconds, before disappearing." While their response states "Regarding the selective loss of LysoTracker-labeled vesicles, we do not expect it to be detectable with such small pool of lysosomes undergoing exocytosis and therefore, do not claim this." So, do the authors see or don't see exocytosis? Further, the claims showing 10% lysosome exocytosis refer to the entire cell surface. But the lysosomes relevant here are the ones near the cell tips. Based on the images and videos presented

© 2021. Published by The Company of Biologists under the terms of the Creative Commons Attribution License (https://creativecommons.org/licenses/by/4.0/).

here form a very small fraction of all the lysosomes in the cells. If only 10% of these lysosomes fuse, then we are talking about just a couple of fusion events, which will not provide the extent of LAMP1 and b-hex release reported here. So, it is clear that the number of exocytic lysosomes have to be >10% and hence should be detected by live imaging assay.

- Neither of the videos present evidence for increased co-localization of Rab11 with LE/lysosomes upon ionomycin stimulation, nor is the exocytosis of any of the vesicles obvious from viewing these videos.

- With the inability to clearly distinguish between LE and Ly, and the revised manuscript claiming both these compartments are affected by this Rab11 pathway, there is a need to revise the manuscript to carefully remove the reference to lysosomes only as it is a source of confusion throughout the text.

- With the revised claim that Rab11 "…interaction is required for the last steps of LE/lysosome exocytosis" the authors should correct this claim throughout the text instead claiming lysosomes at some places and LE/lysosomes at others.

- The data presented in the supplementary figures are still shown as bar graph. These should also be replaced with the dot plots.

Reviewer 2

Advance summary and potential significance to field

In this study, the authors elucidate a role of Rab11a and b in Ca2+-induced lysosomal exocytosis, in cultured HeLa cells. They allude to a transient interaction of Rab11-positive with lysotracker positive vesiclesat cell periphery (that is Ca2+ induced).Sec15,which interacts with Rab11 also appears to affect lysosome exocytosis, although other subunits of the exocyst complex do not appear to play a role. Lastly, the Rab3 GEF, RAB also interacts with Rab11 (forming a potential molecular link between Rab11 and Rab3 positive vesicles). They thus propose an interesting model of a cascade where Rab11 recruits GRAB to cell periphery and this serves to recruit Rab3A for positioning of lysosomes for exocytosis (Rab3 having previously been implicated in this process).

Lysosome exocytosis is a topic of interest and better understanding mechanisms underlying this, is important and worthy of publication.

Comments for the author

The authors have addressed most of the concerns raised by the reviewers including a) the concerns with colocalization (including attempts to use Airyscan imaging to improve their colocalization studies, which it has-F.ig.2C).
b)They have also performed live imaging experiments to examine other members of the exocyst complex to address their lack of involvement in this lysosomal exocytosis process
c)Likewise, they have added qualifying statements where there were concerns of over-interpretation.
d) They have made efforts to perform rescue experiments:while these were unsuccessful and this could be seen as an issue of not establishing "necessity" of the proteins in the process, the reviewer acknowledges that transfection can alter lysosome status and function. They have used several different constructs for depletion to improve confidence on involvement of the molecules.
It might be good to add this statement (regarding the lack of rescue and explanation in the manuscript; this will help other researchers as well, when attempting similar assays).
e)Lastly, they have addressed the issues pointed out with statistics.
In my opinion the paper should be published, with minor revisions [indicated in point (d)].

© 2021. Published by The Company of Biologists under the terms of the Creative Commons Attribution License (https://creativecommons.org/licenses/by/4.0/).

Second revision

Author response to reviewers' comments

Reviewer 1
Comments:

Use of a pool of siGENOME siRNAs does little to establish the specificity of shRNA- mediated knockdown and its rescue by re-expressing the shRNA resistant GFP Rab11a/b. Assuming shRNA resistant GFP-Rab11a/b cDNAs were used for the rescue (were they?), it is evident that these plasmids offer similar level of rescue of LAMP1 and b-hex secretion as GFP only cDNA. To explain this discrepancy, authors claim that "…protein overexpression by themselves induce lysosome secretion, possibly due to the stress caused to the cell by the uptake of the lipid/DNA complexes.". If transfections do indeed alter lysosomal behavior in the experimental conditions used here, then this raises grave concerns regarding the validity of experiments performed throughout the manuscript (e.g. Figures 2, 4, 5, 6, and several supplemental data) that uses transfection to express fluorescently-tagged Rabs. This issue needs to be clearly resolved.

We attempted to perform the rescue experiment suggested by the Reviewer, since we also think it is an important one. For that, we overexpressed mouse Rab11a-GFP or Rab11b-GFP in HeLa cells transduced with shRNAs targeting human Rab11a or Rab11b, respectively, thus avoiding the silencing of the cDNAs expressed. However, as referred in our previous rebuttal letter, we observed an increase in both LAMP1 cell surface translocation and ß-hexosaminidase release in cells silenced for Rab11a or Rab11b and overexpressing GFP. We speculated that "cell transfection and protein overexpression by themselves induce lysosome secretion, possibly due to the stress caused to the cell by the uptake of the lipid/DNA complexes", but have not referred that in the manuscript as we cannot be sure. Whatever the reason, the increase in both LAMP1 cell surface translocation and ß-hexosaminidase release upon GFP overexpression precludes the rescue experiments.

To address if the perceived colocalization of Rab11 and LAMP1-positive vesicles at the cell tips is not due to the vesicles being tightly packed vesicles in a that space, authors carried out live cell imaging of Rab11a with LysoTracker/Dextran 647. While there is no lysotracker data in figure S4, the data shown with dextran 647 again fails to resolve individual dextran-labeled vesicle. So, this data does little to answer the original concern regarding need for greater resolution by using electron microscopy (EM) of super-resolution microscopy of the vesicles at the cell tip. The only effort in this direction has been presented in revised figure 2C showing vesicles adjacent to the perinuclear region where there is no vesicle crowding and no expectation for co- localization as stated that Rab11-positive vesicles "…interact with LEs/lysosomes at the cell periphery just before lysosome fusion with the plasma membrane." Thus, this colocalization in the perinuclear space is puzzling and a clear evidence for co- localization at cell tip is still lacking.

To further elucidate this point and convincingly show that Rab11-positive vesicles interact with LEs/lysosomes labelled with LysoTracker, in particular at the cell tips, we performed high resolution live cell imaging experiments using a Zeiss LSM980 confocal microscope with Airyscan. For this, HeLa cells overexpressing Rab11a-GFP and incubated with LysoTracker for 1-2h were imaged in the absence or immediately after incubation with 4 mM ionomycin and 4 mM $CaCl_2$. Since vesicles at the cell tips are always more closely packed, we imaged cell tips and also other areas of the cell. Moreover, we selected cells with low Rab11a-GFP expression. Several frames were acquired, in intervals of 4-6 sec, for 60-90 seconds, to detect colocalization between Rab11a-GFP-positive vesicles and LEs/lysosomes labeled with LysoTracker. Three independent experiments were performed and □50 cells were analyzed in total. Transient interactions between Rab11a-GFP-positive vesicles and LEs/lysosomes were clearly observed both in the absence and presence of ionomycin. Quantification of the number of interactions observed, using the ICY spot detector plugin, showed a significant increase in the number of interactions between Rab11a-GFP-positive vesicles and LE/lysosomes, immediately after ionomycin stimulation, further supporting

© 2021. Published by The Company of Biologists under the terms of the Creative Commons Attribution License (https://creativecommons.org/licenses/by/4.0/).

our claims (new Figure 2B and C; Videos 1 and 2).

In the manuscript text authors state that "…we postulated that Rab11 and Rab3a interaction is mediated by an adaptor protein. [GRAB]". However, in their response authors contradict themselves by speculating "we postulate that GRAB is not essential for Rab11-positive vesicles to interact with late endosomes/lysosomes, i.e. in the absence of GRAB, Rab11-positive vesicles can still interact with Rab3a-positive late endosomes/lysosomes." This contradiction needs to resolved as this data is critical to the overall model proposed by this study.

We postulate that Rab11a interaction with Rab3a is mediated by GRAB but we do not know if GRAB is essential. In the absence of GRAB, Rab11-positive vesicles and LEs/lysosomes could still interact but Rab3a would not be activated, impairing lysosome exocytosis.

In another contradiction, in the manuscript authors make the claim "Upon ionomycin stimulation, the increase in intracellular calcium concentration leads to colocalization of LysoTracker-positive vesicles with GFP Rab11a-positive vesicles, for a few seconds, before disappearing." While their response states "Regarding the selective loss of LysoTracker-labeled vesicles, we do not expect it to be detectable with such small pool of lysosomes undergoing exocytosis and therefore, do not claim this." So, do the authors see or don't see exocytosis? Further, the claims showing 10% lysosome exocytosis refer to the entire cell surface. But the lysosomes relevant here are the ones near the cell tips. Based on the images and videos presented here form a very small fraction of all the lysosomes in the cells. If only 10% of these lysosomes fuse, then we are talking about just a couple of fusion events, which will not provide the extent of LAMP1 and b-hex release reported here. So, it is clear that the number of exocytic lysosomes have to be >10% and hence should be detected by live imaging assay.

Considering the new results obtained by live cell imaging, we replaced the sentence referred above to "We also observed that LysoTracker-positive LEs/lysosomes transiently interact with Rab11a-GFP-positive vesicles (Fig. 2B, Fig. S4A, Video 1, 2, 3). These events were mostly detected at the cell tips or close to the plasma membrane.", since in some cells LysoTracker-positive LEs/lysosomes disappear upon the transient interaction (Figure 2B, Video 1), but in others remain visible (Figure 2B, Video 2).

Neither of the videos present evidence for increased co-localization of Rab11 with LE/lysosomes upon ionomycin stimulation, nor is the exocytosis of any of the vesicles obvious from viewing these videos

We now have clear high resolution microscopy images showing LysoTracker-positive LEs/lysosomes traveling to the cell periphery and interacting transiently with Rab11a- positive vesicles. In some cells, the LysoTracker-positive vesicles disappear after that, presumably due to the fusion with the plasma membrane (Video 1). We also provide new data showing a significant increase in the number of interactions between Rab11a- positive vesicles and LEs/Lysosomes upon ionomycin stimulation, when compared with the number of interactions detected in the absence of ionomycin (new Figure 2C).

With the inability to clearly distinguish between LE and Ly, and the revised manuscript claiming both these compartments are affected by this Rab11 pathway, there is a need to revise the manuscript to carefully remove the reference to lysosomes only as it is a source of confusion throughout the text.
With the revised claim that Rab11 "…interaction is required for the last steps of LE/lysosome exocytosis" the authors should correct this claim throughout the text instead claiming lysosomes at some places and LE/lysosomes at others.
In the case of ß-hexosaminidase secretion assays, we can refer to lysosome exocytosis. Whenever we only have evidence based on LysoTracker/LAMP-1 staining, we refer to LEs/lysosomes. We verified the manuscript to be consistent with this criterion.

The data presented in the supplementary figures are still shown as bar graph. These should also be replaced with the dot plots.

© 2021. Published by The Company of Biologists under the terms of the Creative Commons Attribution License (https://creativecommons.org/licenses/by/4.0/).

We have replaced the bar graphs of the mRNA expression levels by qRT-PCR by dot plots, as requested by the Reviewer.

Reviewer 2 Comments:
d) They have made efforts to perform rescue experiments: while these were unsuccessful and this could be seen as an issue of not establishing "necessity" of the proteins in the process, the reviewer acknowledges that transfection can alter lysosome status and function. They have used several different constructs for depletion to improve confidence on involvement of the molecules. It might be good to add this statement (regarding the lack of rescue and explanation in the manuscript; this will help other researchers as well, when attempting similar assays).

Following the Reviewer’s suggestion, we have now included in the discussion a sentence concerning this issue (“We attempted to restore lysosome exocytosis in cells depleted for Rab11a or Rab11b by overexpressing the same Rab isoform, but we observed that silenced cells overexpressing GFP display increased levels of both LAMP1 cell surface translocation and ß-hexosaminidase release, thus precluding this approach.”).

Third decision letter

MS ID#: JOCES/2020/246694

MS TITLE: A novel Rab11-Rab3a cascade required for lysosome exocytosis

AUTHORS: Cristina Escrevente, Liliana Bento-Lopes, Jose S. Ramalho, and Duarte C. Barral
ARTICLE TYPE: Research Article

We have now reached a decision on the above manuscript.

To see the reviewers' reports and a copy of this decision letter, please go to: https://submit-jcs.biologists.org and click on the 'Manuscripts with Decisions' queue in the Author Area. (Corresponding author only has access to reviews.)

I asked both reviewers for comments on the latest version of your manuscript, which are appended below. The addition of high resolution imaging data has helped to substantiate the claim that “Rab11-positive vesicles transiently interact with lysosomes at the cell periphery”. However, I agree with reviewer 1 assessment that the â€œdata still remain speculativeâ€•, specifically the claim that Rab11-GRAB-Rab3 cascade regulates lysosome exocytosis. Given the investment of time and the potential interest in the study, Iâ€™m inviting you to revise your manuscript again. I suggest you to carefully read my comments and suggestion for experiments, as described below. Also, I request you to send me a detailed response on how you will approach this revision, if you decide to revise the study again.

I am again defining my major concern, which is also stated in the reviewer #1 comment # 3. The concern remains -How does Rab11 regulate lysosome exocytosis? Does the pathway involve GRAB-Rab3 complex or not? Several statements in the manuscript suggest so, (text on page 11, title of Figure 6 and title of the study) but are not supported by experimental data. The data shown in the manuscript that Rab11 interacts with GRAB and Rab3 does not indicate, â€œRab11-GRAB-Rab3a cascade is required for calcium-dependent lysosome exocytosisâ€•. There are several proof-of-concept experiments missing in the study to support this major conclusion and must be included if you decide to revise the study. For instance, does Rab11 and Rab3 interaction depend on GRAB (as implied by Figure 6 title), if so; please test the interaction in GRAB-depleted cells. An important experiment that could be incorporated is the extent of Rab11 and lysotracker colocalization in GRAB-depleted cells. What are the levels of active Rab3 in Rab11 depleted cells (Rab3 membrane localization upon Rab11 depletion can address this). Does constitutively active Rab3 rescues

© 2021. Published by The Company of Biologists under the terms of the Creative Commons Attribution License (https://creativecommons.org/licenses/by/4.0/).

lysosome exocytosis defect in Rab11 depleted cells (also suggested by the reviewer 1)? What are the levels of active Rab3 in Rab11 depleted cells?

-Regarding pt #2 mentioned by reviewer #1- please address whether the video is a confocal slice or projection in the manuscript text. To strengthen data shown in Figure 2, the authors can think of additional control in Figure 2 such as assessment of colocalization of Rab11 endosomes with lysotracker where motility or positioning of lysosomes is disrupted. Please also address the comment by reviewer 2 on inset image figure 2B.

-Further, please replace the blot shown in Figure 6A and add densitometric quantification of the blot, as the input level of GRAB mutant seems to be quite different, as compared to the WT protein. This concern was also pointed by the reviewer 1 and has not been rectified in the manuscript image. A suggestion is to include data in the figure S6 (reverse co-ip) with the main figure 6.

-Regarding pt #1 of the reviewer, here are my inputs- although addition of rescue experiments is crucial to alleviate the probability of off-target effects, I think that use of five different shRNAs does indicate that the phenotype is unlikely to be an off-target effect.

We are aware that you may be experiencing disruption to the normal running of your lab that makes experimental revisions challenging. If it would be helpful, we encourage you to contact us to discuss your revision in greater detail. Please send us a point-by-point response indicating where you are able to address concerns raised (either experimentally or by changes to the text) and where you will not be able to do so within the normal timeframe of a revision. We will then provide further guidance. Please also note that we are happy to extend revision timeframes as necessary.

Please ensure that you clearly highlight all changes made in the revised manuscript. Please avoid using 'Tracked changes' in Word files as these are lost in PDF conversion.

Please also provide a point-by-point response detailing how you have dealt with the points raised by me and the reviewers in the 'Response to Reviewers' box. If you do not agree with any of their criticisms or suggestions please explain clearly why this is so.

Reviewer 1

Advance summary and potential significance to field

The revised manuscript addressed the previously raised minor comments, but the major comments remain unaddressed. Despite revisions, the data (and many of the claims made) still remain speculative and poorly or unsubstantiated by the data. Below I have reiterated 3 of these major concerns, but this is reflected in other aspects of this work that I have not commented on, e.g., how Sec15 affects LE/Ly exocytosis and its relevance to this study.

Comments for the author

1. Authors' responses that …"cell transfection and protein overexpression by themselves induce lysosome secretion, possibly due to the stress caused to the cell by the uptake of the lipid/DNA complexes", and …"Whatever the reason, the increase in both LAMP1 cell surface translocation and β-hexosaminidase release upon GFP overexpression precludes the rescue experiments.", reiterates my past concern "If transfections do indeed alter lysosomal behavior in the experimental conditions used here, then this raises grave concerns regarding the validity of experiments performed throughout the manuscript" I see authors prefer to dismiss this concern, rather than address it.

2. Regarding live imaging for exocytic lysosomes, the new video (not stated if it's a confocal slice or projection; latter would preclude claimed co-localization) shows LE/Ly either remain static or move (split, disappear, or disappear and reappear) throughout the cell. It does not substantiate claims regarding LE/Ly fusion, or interaction of Rab11 vesicle with fusing LE/Ly, or that these occur

© 2021. Published by The Company of Biologists under the terms of the Creative Commons Attribution License (https://creativecommons.org/licenses/by/4.0/).

at cell tips. Further, with 1% Rab11 vesicles interacting with LE/Ly (in a confocal slice or 3D projection?) it is not feasible to determine how this relates to the overall magnitude of surface LAMP1 or enzyme release?

3. A major claim of this study is the role of GRAB in regulating lysosome exocytosis. However, in response to my last comment regarding contradictory statements on the role of GRAB in the Rab11 interaction with LE/Ly, authors have offered additional speculation, instead of addressing GRAB's role. So, the need for clear evidence to support the role of Rab11, GRAB, Rab3 complex in lysosome fusion remains and still requires additional experiments. If GRAB is required for just activating Rab3, then the need for GRAB and Rab11 should be circumvented by constitutively active Rab3. Such experiments can shed light on the mechanism that is central to the claim from this study.

Reviewer 2

Advance summary and potential significance to field

In this study, the authors elucidate a role of Rab11a and b in Ca2+-induced lysosomal exocytosis, in cultured HeLa cells. They allude to a transient interaction of Rab11-positive with lysotracker positive vesicles at cell periphery (that is Ca2+ induced).Sec15,which interacts with Rab11 also appears to affect lysosome exocytosis, although other subunits of the exocyst complex do not appear to play a role.
Lastly, the Rab3 GEF, RAB also interacts with Rab11 (forming a potential molecular link between Rab11 and Rab3 positive vesicles). They thus propose an interesting model of a cascade where Rab11 recruits GRAB to cell periphery and this serves to recruit Rab3A for positioning of lysosomes for exocytosis (Rab3 having previously been implicated in this process).

Lysosome exocytosis is a topic of interest and better understanding mechanisms underlying this, is important and worthy of publication.

Comments for the author

The authors have addressed concerns raised by the reviewers. They have added statements to qualify their findings including adding in statements about why the rescue experiments did not work as may have been expected. They have also carried out additional super resolution experiments (airy scan imaging) that allows for imaging at high temporal and spatial resolution simultaneously. These studies and quantitative analysis from those images strengthen the point being made regarding Rab11 and lysotracker-positive vesicle interactions.
There is some pixelation in figure 2 B insets that have been enlarged (but this is most likely to do with post acquisition processing and should be relatively easy to fix)-they also don't detract/change the message conveyed.

Third revision

Author response to reviewers' comments

For instance, does Rab11 and Rab3 interaction depend on GRAB (as implied by Figure 6 title), if so; please test the interaction in GRAB-depleted cells.

We attempted to uncover if GRAB is essential for Rab11-Rab3a interaction. For that, we immunoprecipitated endogenous Rab11a and detected Rab3a by Western-blot in HeLa cells silenced for GRAB. Since the interaction between Rab11 and Rab3 is not robustly detected with endogenous levels of these proteins, we co-immunoprecipitated overexpressed mCherry-Rab11a and GFP-Rab3a in HeLa cells silenced for GRAB. However, the percentage of cells that overexpress both mCherry-Rab11a and GFP- Rab3a is small. Furthermore, the transfection efficiency and

© 2021. Published by The Company of Biologists under the terms of the Creative Commons Attribution License (https://creativecommons.org/licenses/by/4.0/).

overexpression levels are different in cells silenced for GRAB or control cells and we could not normalize the differences in the interaction to the amount of overexpressed proteins.

Additionally, we tried to use purified proteins, namely His-Rab11a, GST-Rab3a and GRAB-MBP. First, we confirmed that GRAB interacts with Rab11, as described in the literature. Then, we tested Rab11a-Rab3a interaction. For this, we immunoprecipitated GST-Rab3a using Glutathione Sepharose beads or His-Rab11a using Protein G Sepharose beads, but in both cases we observed unspecific binding of the co- immunoprecipitated proteins to the beads alone control. We adjusted our experimental conditions by blocking the unspecific binding with BSA, preclearing the protein mixture with beads alone and increasing the amount of salt and detergent of the washing buffers. However, these optimizations failed to give consistent results. Finally, we tested the interaction between Rab11a-Rab3a when GRAB was present or not, but the results were not reproducible. It is possible that the tags preclude the protein interactions by causing steric hindrances.

Although our results confirm that GRAB interacts with Rab11a and GRAB is a known Rab3a GEF, we could not convincingly demonstrate that GRAB is essential for Rab11a- Rab3a interaction. Therefore, we changed the claims in the manuscript and the legend on Figure 6.

An important experiment that could be incorporated is the extent of Rab11 and lysotracker colocalization in GRAB-depleted cells.

Our live cell imaging results show that Rab11-positive vesicles transiently interact with LysoTracker-positive vesicles at steady state and that these interactions increase significantly upon ionomycin stimulation. Nevertheless, the interactions observed are few, since they are transient. Technically, the suggested experiment has several obstacles: we cannot be sure of the cells that are silenced for GRAB or not and to what extent each cell is silenced; only some transfected cells express GFP-Rab11a; and transfected cells tend to have a lower number of LysoTracker-positive vesicles and these tend to be more perinuclear, probably due to the effect of transfection on lysosome exocytosis discussed in the first resubmission. All these preclude the proposed approach.

What are the levels of active Rab3 in Rab11 depleted cells (Rab3 membrane localization upon Rab11 depletion can address this).

We hypothesize that only Rab3a recruited to lysosomes becomes activated by GRAB. However, this represents a very small percentage of the Rab3a present in the cell. Indeed, most of Rab3a localizes to the Golgi in steady-state and only a small percentage to lysosomes. Therefore, analyzing Rab3a activity in the total cell lysates will not answer this question and experimentally, it is not feasible to measure the activation levels of Rab3a specifically in lysosomes.

Furthermore, we hypothesize that GRAB, transported by Rab11 is required for Rab3a activation but not for Rab3a localization. Consequently, in the absence of Rab11 or GRAB, Rab3a localization should not be affected.
Since we could not show that GRAB recruitment by Rab11 is required for Rab3a activation, we removed this claim from the manuscript. We now only suggest this as an hypothesis.

Does constitutively active Rab3 rescues lysosome exocytosis defect in Rab11 depleted cells (also suggested by the reviewer 1)?
As discussed previously, transfected cells show increased levels of lysosome exocytosis, precluding the discrimination between an effect of the constitutively active form of Rab3a from the effect of the transfection itself.

Regarding pt #2 mentioned by reviewer #1- please address whether the video is a confocal slice or projection in the manuscript text.
The video shown in Figure 2 is a Z-plane and not a projection. This information was added to the Figure legend.

To strengthen data shown in Figure 2, the authors can think of additional control in Figure 2 such as assessment of colocalization of Rab11 endosomes with lysotracker where motility or positioning of lysosomes is disrupted.

© 2021. Published by The Company of Biologists under the terms of the Creative Commons Attribution License (https://creativecommons.org/licenses/by/4.0/).

As discussed above, colocalization between Rab11-positive vesicles and LysoTracker- positive vesicles is limited as the interactions are transient. Moreover, upon cell transfection, we detect less LysoTracker-positive vesicles and these tend to be more perinuclear. Therefore, this phenotype is already what is expected from disrupting lysosome motility, for example by silencing Arl8b.

Please also address the comment by reviewer 2 on inset image figure 2B.

We provide a less pixelated image in the new version of Figure 2.

Further, please replace the blot shown in Figure 6A and add densitometric quantification of the blot, as the input level of GRAB mutant seems to be quite different, as compared to the WT protein. This concern was also pointed by the reviewer 1 and has not been rectified in the manuscript image. A suggestion is to include data in the figure S6 (reverse co-ip) with the main figure 6.
Figure 6A was exchanged by Figure S6A and densitometric quantification added.

Fourth decision letter

MS ID#: JOCES/2020/246694

MS TITLE: Rab11 is required for lysosome exocytosis through the interaction with Rab3a, Sec15 and GRAB

AUTHORS: Cristina Escrevente, Liliana Bento-Lopes, Jose S. Ramalho, and Duarte C. Barral
ARTICLE TYPE: Research Article

I am happy to tell you that your manuscript has been accepted for publication in Journal of Cell Science, pending standard ethics checks.

© 2021. Published by The Company of Biologists under the terms of the Creative Commons Attribution License (https://creativecommons.org/licenses/by/4.0/).
